# Rationalised experiment design for parameter estimation with sensitivity clustering

**DOI:** 10.1038/s41598-024-75539-2

**Published:** 2024-10-28

**Authors:** Harsh Chhajer, Rahul Roy

**Affiliations:** 1 Department of Bioengineering, Indian Institute of Science, Bangalore, 560012 India; 2 Department of Chemical Engineering, Indian Institute of Science, Bangalore, 560012 India

**Keywords:** Approximate Bayesian computation, Model fitting, Informative experiment design, Parameter sensitivity, Clustering-based experiment design, Computational biology and bioinformatics, Computational platforms and environments, Statistical methods, Biomedical engineering, Microbiology

## Abstract

Quantitative experiments are essential for investigating, uncovering, and confirming our understanding of complex systems, necessitating the use of effective and robust experimental designs. Despite generally outperforming other approaches, the broader adoption of model-based design of experiments (MBDoE) has been hindered by oversimplified assumptions and computational overhead. To address this, we present PARameter SEnsitivity Clustering (PARSEC), an MBDoE framework that identifies informative measurable combinations through parameter sensitivity (PS) clustering. We combined PARSEC with a new variant of Approximate Bayesian Computation-based parameter estimation for rapid, automated assessment and ranking of experiment designs. Using two kinetic model systems with distinct dynamical features, we show that PARSEC-based experiments improve the parameter estimation of a complex system. By its inherent formulation, PARSEC can account for experimental restrictions and parameter variability. Moreover, we demonstrate that there is a strong correlation between sample size and the optimal number of PS clusters in PARSEC, offering a novel method to determine the ideal sampling for experiments. This validates our argument for employing parameter sensitivity in experiment design and illustrates the potential to leverage both model architecture and system dynamics to effectively explore the experimental design space.

## Introduction

The scientific approach relies on carefully designed experiments to understand complex systems. When analysing a system, researchers must decide which variables to measure, as well as when, where, and how to conduct these measurements, which collectively form the experimental design. The set of all possible choices forms the design space. A well-constructed experiment should offer quantitative data for precise model parameter estimation and model selection, which provides a better understanding of the system and improves model predictions. For this purpose, the experimental design should be both cost-effective and feasible, optimising accuracy and information yield while reducing the number of measurements. However, the increasing complexity and sophistication of systems make it challenging to rely solely on intuition for experimental design.

Compared to traditional model-free techniques, Model-Based Design of Experiments (MBDoE) algorithms use system-specific models to optimise experiment design, leading to improved parameter estimation^1–3^. By leveraging these models, MBDoE enables a more effective evaluation of the experimental design space, providing a comprehensive understanding of the variables and their interactions. This, in turn, can facilitate resource optimisation, reduce the number of trials, and improve the accuracy of model parameter estimation^2,3^ and model selection^4^. Over the years, MBDoE has been widely applied in a variety of fields, including engineering and biomedicine, due to the growing use of quantitative models to understand complex systems. However, despite its advantages, the application of MBDoE in parameter estimation is hindered by the increasing complexity of modern experiments and their inherent constraints.

Fisher’s Information Matrix (FIM) is a popular MBDoE framework for designing experiments aimed at estimating model parameter values^2–5^. It is based on optimising properties (based on trace, determinant) of the inverse of the FIM suggesting experiment designs for parameter identifiability, estimation precision and accuracy. The elements of the FIM are calculated by taking the expected value of the second derivative of the log-likelihood function with respect to the model parameters^6,7^. If changes in the values of the parameters have a major effect on the output of the model, it implies that the output is useful in providing information about the parameters. FIM-MBDoEs typically rely on a linear statistical model to relate measurements to parameter values, which requires linearising the nonlinear model equations at expected ground-truth parameter values^8,9^. However, when these guesses are inaccurate, the efficiency of the experiment designs is significantly compromised, limiting the ability of FIM methods to account for parameter uncertainty in experimental design^6^. Furthermore, the complexity of matrix inversion and the need for a large experimental sample size to achieve the desired accuracy and a Gaussian noise distribution present additional difficulties that restrict the use of FIM-MBDoE^10–13^. To address these limitations, recent studies have proposed new approaches such as experiment design space discretisation to handle non-linear models, and calculating the average information content and risk for large uncertainties to improve experiment designs^5–7,10,14–16^. Additionally, the pseudo-inverse^17,18^ can be used to tackle ill-conditioned matrices^5^.

Alternately, Approximate Bayesian approaches to MBDoE offer a distinct advantage by operating independently of assumptions related to linearity or direct likelihood estimations^19–25^. These techniques can take into account any prior information or convictions about the parameters and offer more flexibility in accounting for parameter uncertainty. Nonetheless, these methods depend on approximating the likelihood function and the utilization of summary statistics, rendering them sensitive to the selected statistics. Furthermore, they are computationally demanding, particularly when dealing with complex systems or large experiment design spaces, and may produce biased and sub-optimal experiment designs when tailored to pre-specified sample sizes^20–22^. To address these limitations, certain algorithms employ a sequential greedy approach, which builds on existing experiment designs to manage complexity and improve design efficiency. However, such design search approaches are influenced by the initial design choice and may be susceptible to local optima artifacts.

To address these limitations, we introduce the PARSEC (PARameter SEnsitivity-driven Clustering-based) DoE algorithm, which exploits the model equations describing the coupling of system variables through parameter sensitivity analysis to direct the search for informative experiment designs. PARSEC works by identifying measurement time combinations with distinct parameter sensitivity indices (PSI) vector representations. These PSIs capture the impact of model parameter changes on measurable output variables, indicating the measurements that are most sensitive to dynamical changes in the system. By clustering PSIs based on Euclidean distance, we can identify a minimal measurement set that captures the essential dynamics enabling precise parameter estimation through better DoE. We show that clustering the PSI vectors and picking a representative from each cluster permits a judicious sampling of the dynamics profile, enhancing the accuracy of parameter estimates and thereby enabling PARSEC to create an ‘optimal’ DoE efficiently. We find that the optimal number of PSI clusters is in agreement with the experiment designs that yield the highest information gain, thus introducing a new way to calculate the sampling frequency.

PARSEC offers several advantages over existing methods. Notably, we demonstrate that employing fuzzy (c-means) clustering of PSI can significantly reduce the number of evaluations required to identify optimal experiment designs. By integrating PSI evaluations at different parameter values, PARSEC facilitates the identification of generalist and robust designs that accommodate prior knowledge uncertainty. Furthermore, we demonstrate the adaptability of PARSEC to address practical and technical considerations, as detailed in the “Methods” section. This approach effectively combines concepts from FIM-based and Bayesian MBDoEs to determine the most effective experiment designs. We demonstrate the flexibility of the PARSEC algorithm by using it on two biological kinetic models with distinctly different dynamic characteristics (specifically oscillatory and saturating kinetics) to determine their best experimental designs. Specifically, we determine the combinations of time points at which model variables should be measured to estimate accurate model parameters.

A reliable and high-throughput parameter estimation framework is needed to effectively implement PARSEC to evaluate the predicted experimental designs. Existing methods for parameter estimation are limited by their inherent assumptions or are not well suited for high-throughput analysis. For instance, likelihood-based techniques are limited to systems where the likelihood function can be specified and a Gaussian distribution can be assumed for parameter distribution and measurement noise^26–28^. In contrast, Approximate Bayesian Computation (ABC) based methods^28–31^ typically rely on data-dependent stipulations (to set error thresholds) or correlated sampling, which can render the estimation vulnerable to local minima and initial biases. To address this, we employ the recently developed Approximate Bayesian Computation - Fixed Acceptance Rate (ABC-FAR) method^32^ for parameter estimation for PARSEC designs. Our ABC-based algorithm uses a global and relative parameter sampling rejection criterion (FAR) that avoids data-dependent stipulations, making it suitable for automated analysis and fair comparison of estimations derived from different data sets. We show that parameter estimation via ABC-FAR is accurate, free of sampling artifacts, and less susceptible to data noise and initial guess bias. Consequently, the implementation of ABC-FAR in PARSEC allows the rapid and robust design of informative experiments.

## Results

### Parameter sensitivity driven design of experiments

**Fig. 1:**
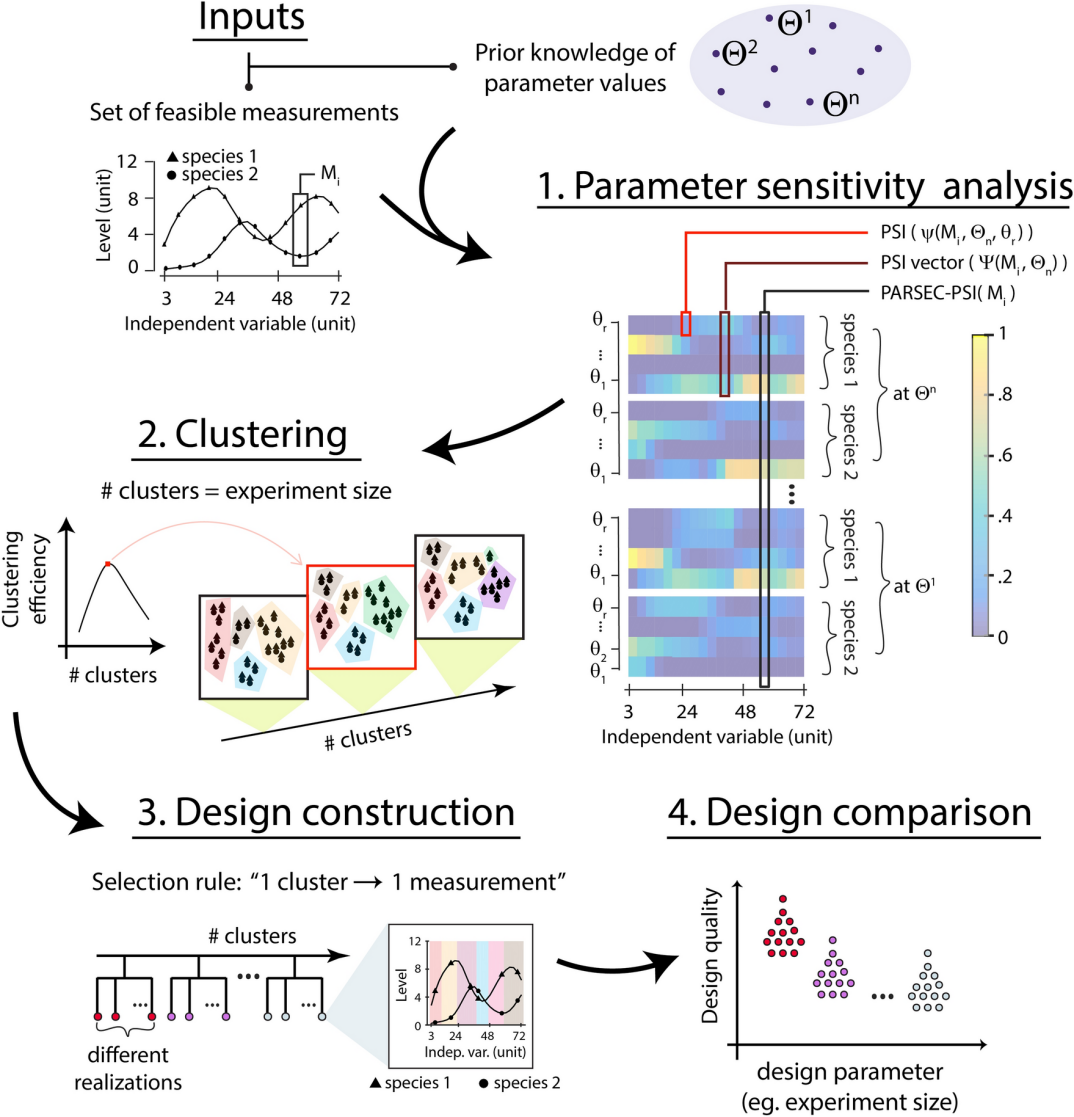
Schematic of PARameter-SEnsitivity Clustering (PARSEC) based experiment design algorithm. PARSEC selects some representative parameter values over the uncertainty range ($\Theta ^k$) and evaluates the parameter sensitivity indices (PSI) vector for every feasible (time point) measurement option (referred as $M_i$, feasibility is determined by the constraints of the experiment) (Step 1). In the schematic, the experiment design involves simultaneous measurements of species 1 and 2 forming a single measurement candidate, so PSI vectors for both species are evaluated. The PSI vectors are evaluated at different parameter values and are vectorially conjoined to create the PARSEC-PSI vector of each measurement candidate (PARSEC-PSI ($M_i$); also see Supplementary SI S1 and Supplementary Fig. S1). Based on the similarity among these PARSEC-PSI vectors, the measurement candidates are clustered using their Euclidean distance (step 2). A candidate from each cluster is randomly selected to form the predicted design (step 3). The number of clusters used in partitioning is based on the sample size (number of measurement candidates to be selected) of the experiment, which can also be optimised performing this analysis with varying cluster numbers. PARSEC delivers multiple experiment design realisations can be computationally compared to identify the best designs (step 4). For example, we use ABC-FAR to evaluate the quality of designs based on the accuracy of parameter estimation.

The PARSEC DoE framework is based on four main ideas (Fig. 1). First, it uses the parameter sensitivity of a variable to determine its informativeness for estimating the values of the corresponding parameters^7,33^. This involves computing a vector of parameter sensitivity indices (PSI) for each variable to be measured (Step 1, Fig. 1). Second, the PSI vector computation is performed with multiple parameter values sampled to cover the distribution associated with parameter uncertainty. The PSI vectors across different parameter values for a measurement candidate are vectorially conjoined to yield the final PARSEC-PSI vector which incorporates parameter uncertainty and increases the robustness of DoEs (see Supplementary SI S1, Supplementary Fig. S1). Third, PARSEC selects measurements that minimise overlap (or maximise Euclidean distance) between their associated PARSEC-PSI vectors, maximising the total information obtained from their combination given a fixed sample size. This is achieved by partitioning the measurements based on their PARSEC-PSI vectors and selecting a measurement from each cluster (Step 2, Fig. 1). The effectiveness of the PSI-clustering-based experiment design depends on the quality of the clustering, which is determined by the number of clusters that ultimately dictates the sample size. Optimal clustering can be used to refine the sample size, leading to efficient experiment designs that are both cost-effective and informative (Step 3, Fig. 1). The choice of clustering algorithm depends on the experiment design requirements, with k-means and c-means clustering algorithms being suitable for pre-defined sample sizes and high-dimensional, continuous data. This study focuses on two implementations of PARSEC, namely PARSEC(k) and PARSEC(c), which use k-means and c-means clustering algorithms, respectively (see “Methods”).

Finally, the PARSEC framework compares the multiple generated designs (each representing a combination of measurement time points) to identify the optimal ones. This approach alleviates issues arising from the stochastic nature of clustering algorithms and subsequent measurement selection. Among the various objectives that can be used to select ’better’ experiment designs, we chose to compare the PARSEC designs for their ability to report on the model parameter estimates from the measurements. To efficiently compare the predicted designs for estimation accuracy, PARSEC requires an accurate and high-throughput parameter estimation algorithm (Step 4, Fig. 1). To achieve this, we employ the Approximate Bayesian Computation-Fixed Acceptance Rate (ABC-FAR) technique, adapted from our earlier study^32^. This likelihood-free approach enables the automation of parameter estimation, allowing us to evaluate the expected accuracy of parameter estimation for each design and compare them.

**Fig. 2:**
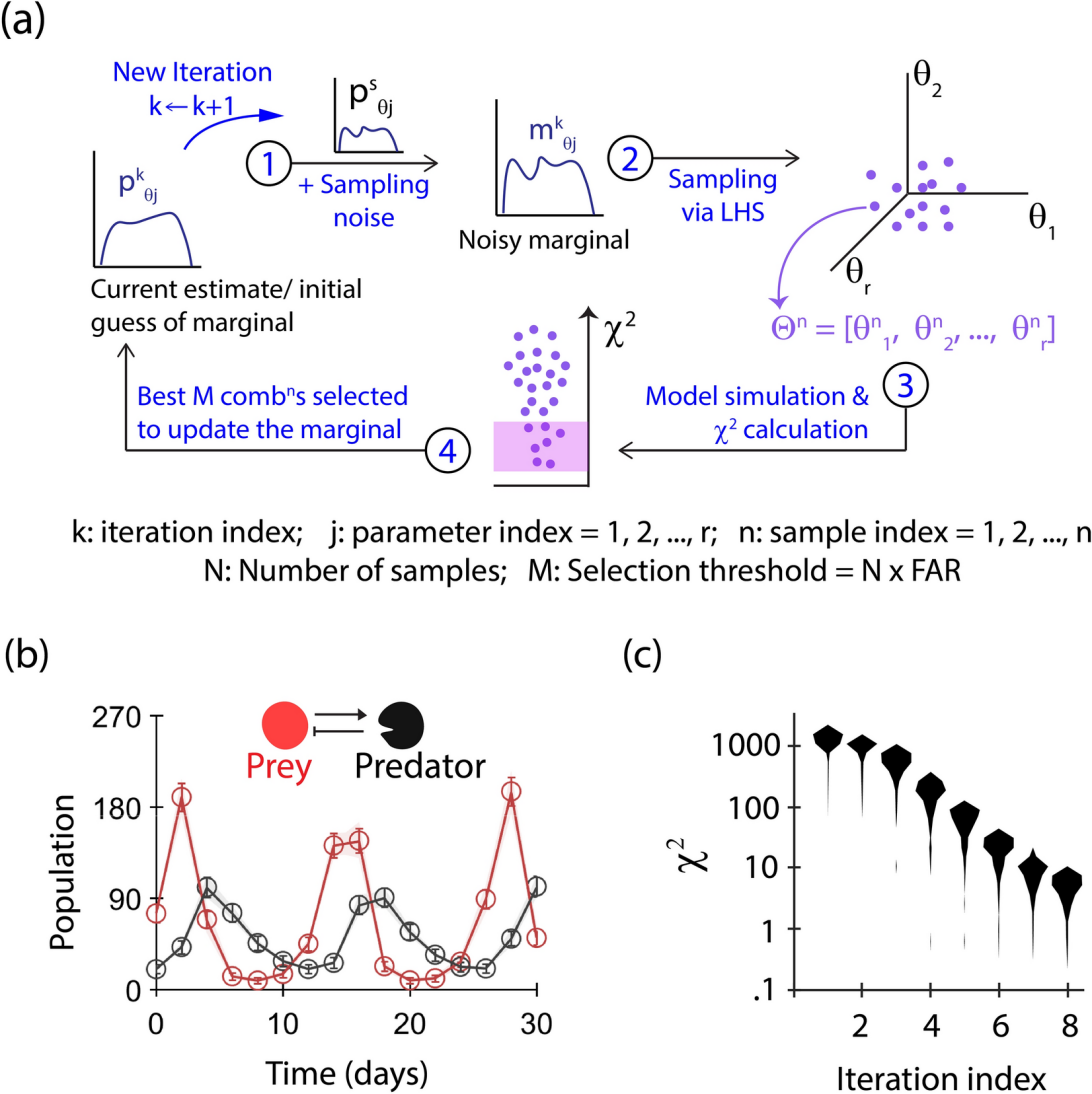
Schematic and performance of ABC-FAR. (**a**) ABC-FAR uses an iterative, ABC approach to estimate the likely distribution of model parameter values that minimises the $\chi ^2$ function (see “Methods” and Supplementary SI S2). Each iteration comprises of the following: (1) ABC-FAR augments the current marginal for each model parameter with a sampling noise. (2) Then, it samples N parameter combinations using Latin Hypercube Sampling^35^. (3) $\chi ^2$ function is evaluated for all samples. (4) M (=FAR $\times$ N) combinations out of the above with the lowest $\chi ^2$ values are used to update the marginal. (**b**) The figure illustrates the results of fitting the Lotka Volterra model to simulated data using ABC-FAR. The solid lines represent the average of the model predictions from the parameter combinations selected by ABC-FAR. The range of the predicted dynamics is indicated by a lighter shade in the background. Since the individual model predictions are very similar, they appear indistinguishable from their average in the figure. The open circles represent the data set used for fitting. For this set, a FAR value of 0.25 and the history-dependent update strategy (see “Methods”) were employed. (**c**) The distribution of $\chi ^2$ values corresponding to the parameter combinations selected in each of the eight iterations is also shown, demonstrating how ABC-FAR reduces $\chi ^2$ iteratively.

Our ABC-FAR method iteratively refines the parameter value distribution using $\chi ^2$ statistics (Fig. 2a). We choose the popular $\chi ^2$ statistics as it serves as a reasonable goodness-of-fit estimator^34^. In each iteration, ABC-FAR samples the current distribution estimate (step 2, Fig. 2a) and selects a subset based on the corresponding $\chi ^2$ values to update the distribution (steps 3 & 4, Fig. 2a). ABC-FAR is distinct from other ABC techniques in that it chooses a fixed proportion (FAR) of the sampled parameter values with the least $\chi ^2$ values to modify the marginals. This criterion eliminates the need for absolute $\chi ^2$ limits, and adjustments specific to the data. Unlike other approaches, ABC-FAR performs a global comparison, ranking all combinations of parameters sampled in an iteration based on the $\chi ^2$ value. We employ Latin Hypercube Sampling (LHS^35,36^) to sample the marginal of each model parameter, eliminating the need for transition kernels. Additionally, noise is added to the sampling distribution to make it more resilient against local minima traps and other biases that may be present in the initial guess (step 1, Fig. 2a).

### Accurate and robust parameter estimation using ABC-FAR

**Fig. 3:**
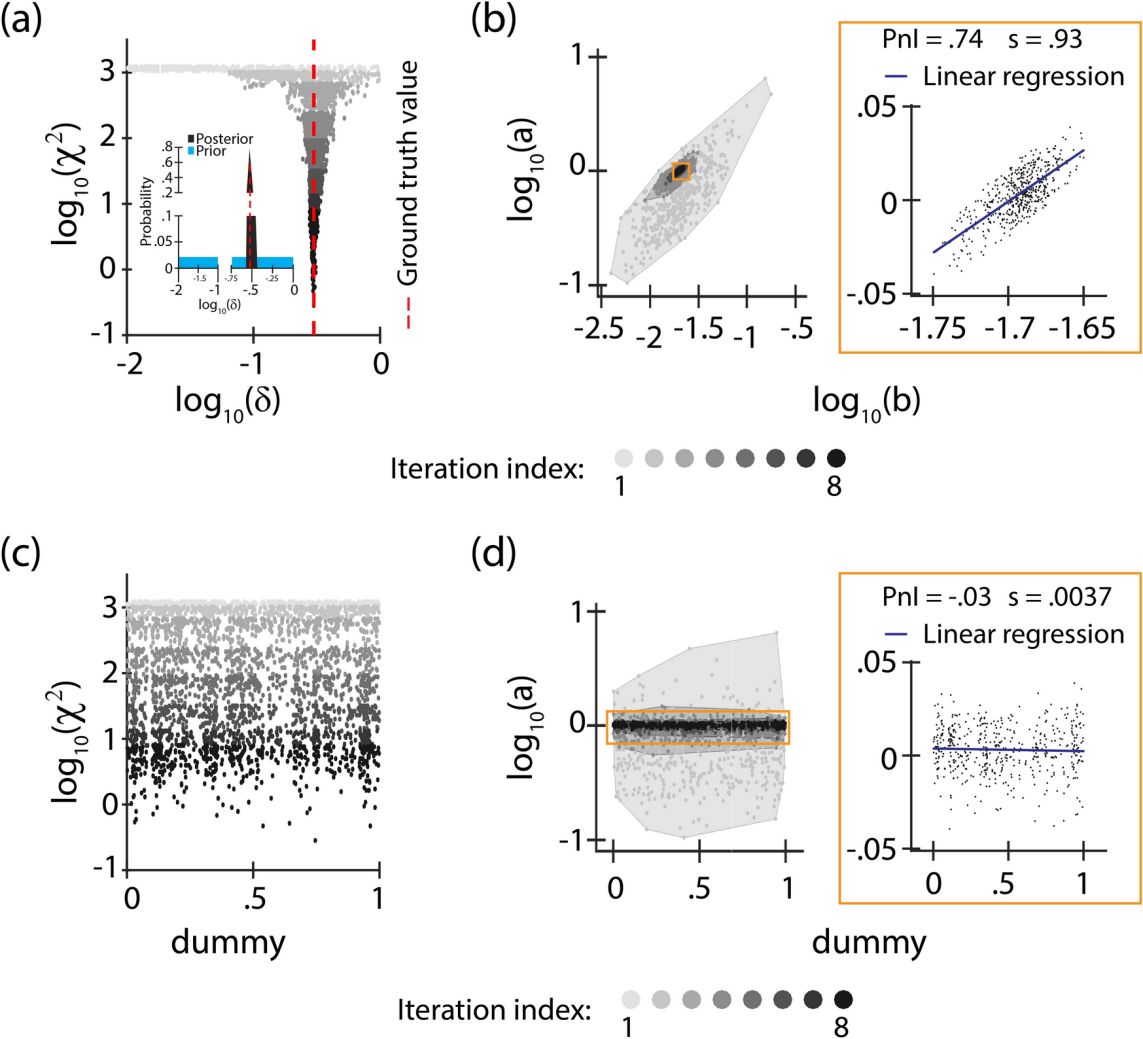
Accuracy and efficiency of parameter estimation with ABC-FAR. (**a**) Plot shows the distribution of the death rate of predators ($\delta$) in a Lotka-Volterra model along with the $\chi ^2$ value corresponding to the parameter combinations selected in each iteration during the fitting of the data-set in Fig. 2b. The inset shows the initial guess (prior) and the estimated posterior for $\delta$. The posterior converges to a sharp distribution around the ground truth value (red dashed line). (**b**) The values of the birth and death rates of prey (*a* and *b* respectively) corresponding to the parameter combinations selected in each iteration of ABC-FAR are shown. The correlation approximates the practical non-identifiability, and the regression (inset) is a measure of the compensatory relation between the two model parameters to keep the $\chi ^2$ constant. (**c**) Plot of the dummy parameter with the $\chi ^2$ value for the same ABC-FAR fitting shows no convergence. The absence of any structure in the selected dummy parameter values indicates no sampling bias in ABC-FAR. (**d**) The value of *a* and the dummy parameter corresponding to the selected combinations are shown. The correlation and regression (inset) measures associated with dummy parameter identify thresholds for statistical significance.

Here, we demonstrate the performance of ABC-FAR using the popular deterministic Lotka-Volterra model (details in Supplementary SI S3). To validate our algorithm for parameter estimation, we adopt a common approach^30^. We create a synthetic data set by simulating the model with a known combination of parameter values (referred to as ground truth) and initial conditions (details in Supplementary SI S4 and Supplementary Tables S1 and S2). Then, with the same initial conditions, we use ABC-FAR to estimate the parameter values for this dataset. We estimate parameter values on a logarithmic scale (base 10) to cover a large dynamic range. We start with a uniform initial guess for all parameters to test performance (Supplementary Table S1). We observe that the difference between the model predictions and the data (the $\chi ^2$ statistic) decreases with each iteration of ABC-FAR, and the model predictions accurately reflect the data (Fig. 2b,c). The algorithm progressively transforms the estimate of the model parameters into a narrow distribution around the corresponding true parameter value (Fig. 3a, Supplementary Fig. S2). However, the marginal of the dummy parameter (see “Methods”, Fig. 3c) remains unchanged, suggesting that the algorithm does not introduce unwanted computation artifacts.

ABC-FAR is also capable of detecting higher-order relationships among the values of the model parameters based on the data. For instance, a strong positive correlation (PRCC = .74) was observed between the ‘birth rate of prey’ (*a*) and ‘death rate of the prey due to predation’ (*b*) (PRCC = .74, Fig. 3b), suggesting poor practical identifiability. The slope of linear regression (=0.93) suggests that changes in $\chi ^2$ due to a 1% increase in $\hbox {log}_{{10}}$(a) value can be compensated by .93% increase in the value $\hbox {log}_{{10}}$(b). The correlations are conditioned on the values of other model parameters, and are statistically significant as is evident when comparing this with a similar analysis on the dummy parameter (Fig. 3d, Supplementary Figs. S3 and S4). We further confirmed that the ABC-FAR-based estimation is robust to measurement noise (Supplementary Fig. S6). Furthermore, ABC-FAR can be adapted to take advantage of the model structure and system-specific information to speed up and improve convergence in parameter estimation (Supplementary SI S7, Supplementary Fig. S7). We have previously demonstrated the ability of the ABC-FAR method to highlight practical identifiability issues in a  more complex model^32^. In that study, we used a generalised dynamical model for positive-sense RNA viruses to identify parameter correlations, and their temporal dynamics, and characterise parameter non-identifiability with ABC-FAR. This suggests the ability of ABC-FAR to be employed beyond the simpler model systems presented in this work.

In our previous analysis, we used a FAR value of 0.25. Choice of FAR value has a distinct effect on ABC-FAR convergence and efficiency. When we compare performance with the estimation accuracy (inversely related to the $$\chi ^2$$ statistics) and cumulative computational cost ($$\propto$$$$\hbox {EAR}^{-1}$$), the $$\chi ^2$$ statistics decreases with the number of iterations, as expected with the ‘history-dependent update strategy’ (see “Methods” and Supplementary Figs. S8–S10). However, the estimation converges to a higher accuracy (i.e., lower $$\chi ^2$$ statistics) as the FAR value decreases. Among those achieving a certain level of accuracy, the schemes with higher FAR values (weaker rejection criteria) appear to be more effective. Nevertheless, the efficiency of our scheme is comparable to that of the popular alternate, the ABC-SMC algorithm^30^ (Supplementary Fig. S10).

### Identifying informative experiment designs using PARSEC with ABC-FAR

We use PARSEC(k) (PARSEC with k-means clustering) and ABC-FAR to design experiments to characterise the parameters of a three-gene repressilator model^37^ (details in “Methods” and Supplementary SI S3). First, we demonstrate the method with predetermined initial conditions and sample size, assuming accurate prior knowledge of parameters. We account for practical considerations, such as (i) limiting the experiment duration, (ii) preferring simultaneous measurements when multiple variables are observed, and (iii) constraining the measurables based on feasibility (see “Methods”).

**Fig. 4:**
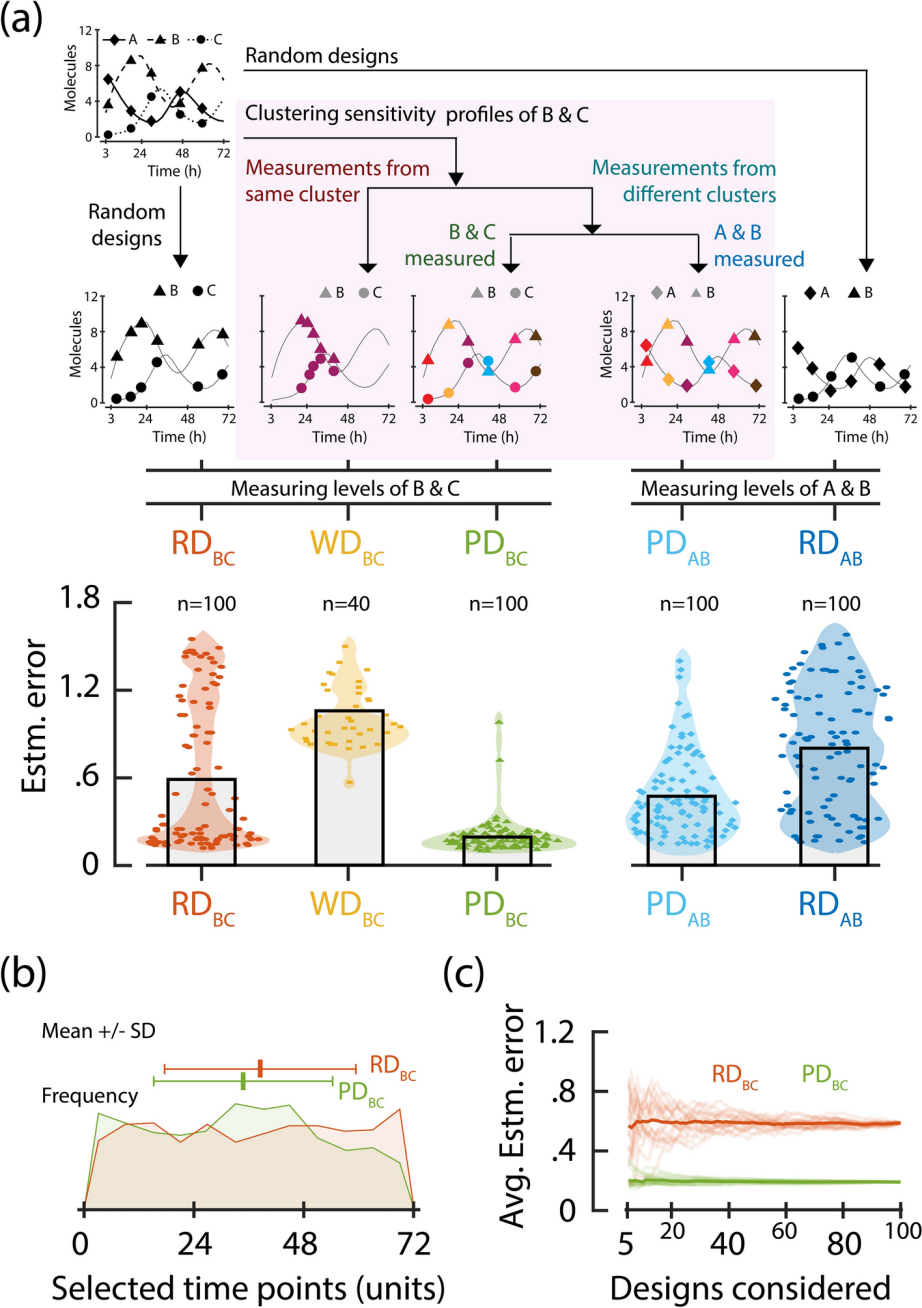
Performance of PARSEC-predicted designs. (**a**) Schemes for the three experiment design algorithms—random, PARSEC and anti-PARSEC—are illustrated (top panel). Random Designs ($\hbox {RD}_{{BC}}$ and $\hbox {RD}_{{AB}}$) are selected in a non-repeating random fashion, whereas the others ($\hbox {PD}_{{BC}}$, $\hbox {WD}_{{BC}}$, and $\hbox {PD}_{{AB}}$) are based on the sensitivity profiles of proteins B and C. For $\hbox {PD}_{{BC}}$ and $\hbox {PD}_{{AB}}$, one and only one measurement candidate is chosen from each cluster (PARSEC approach); while for $\hbox {WD}_{{BC}}$, all measurement candidates are taken from the same cluster. $\hbox {PD}_{{BC}}$, $\hbox {WD}_{{BC}}$ and $\hbox {RD}_{{BC}}$ denote experiment designs where proteins B and C are measured simultaneously (some instances are listed in Supplementary Table S4), while $\hbox {PD}_{{AB}}$ and $\hbox {RD}_{{AB}}$ involve simultaneous measurement of protein A and B. The bottom panel displays the parameter estimation error corresponding to the different designs generated. The bar graphs represent the mean estimation error for each design algorithm. (**b**) The distributions of the different time points selected across the different realisations of $\hbox {PD}_{{BC}}$ (green) and of $\hbox {RD}_{{BC}}$ (red) are shown. These distributions, discretised into 12 equal-length bins, have comparable entropy ($\hbox {PD}_{{BC}}$: 3.55 bits; $\hbox {RD}_{{BC}}$: 3.58 bits), closely resembling that of a corresponding uniform distribution (3.59 bits). The dot and whisker plots above the distributions show the mean and standard deviation. (**c**) Twenty sub-samplings of the set of the predicted experiment designs were considered and the average estimation error across the elements of each set is plotted as a function of the sub-sampling size. The thick lines show the mean behaviour across the sub-samplings.

Here, we design experiments where the levels of proteins B and C can be measured. So, their sensitivity profiles inform the PARSEC design (see “Methods” and SI S3). We evaluated the performance of PARSEC(k) designs ($\hbox {PD}_{{BC}}$) by comparing them with random designs (constructed by a random selection of a set of six measurement time points, labeled as $\hbox {RD}_{{BC}}$) in terms of their accuracy in parameter estimation (subscripts in the label indicate the variables being measured). In addition, we evaluated the significance of clustering by using anti-PARSEC designs ($\hbox {WD}_{{BC}}$) where all measurements are taken from a single cluster (the one with the highest number of candidates). The parameter estimation error for random designs ($\hbox {RD}_{{BC}}$ and $\hbox {RD}_{{AB}}$) is distributed uniformly (Fig. 4a). However, the spread of errors becomes strongly skewed when we consider experiment designs based on PSI vector clustering ($\hbox {PD}_{{BC}}$ and $\hbox {WD}_{{BC}}$, Fig. 4a and Supplementary Fig. S11). Designs, where all the measurements belong to a single cluster (anti-PARSEC, $\hbox {WD}_{{BC}}$), tend to have higher errors, while PARSEC designs ($\hbox {PD}_{{BC}}$) generated using measurements from different clusters have low errors. The top ten percentile of designs for monitoring protein levels of B and C ($\hbox {RD}_{{BC}}$, $\hbox {WD}_{{BC}}$ and $\hbox {PD}_{{BC}}$) are predominantly predicted by PARSEC ($\hbox {PD}_{{BC}}$).

Besides the oscillatory behavior of the repressilator network, we utilised the PARSEC algorithm to design experiments for estimating model parameters in a biological system that exhibits saturating dynamics. To achieve this, we examined a recently published viral life cycle model^32^ that describes the dynamics of six viral molecules (RNA, proteins, and virus titre) with eleven parameters during virus growth in cells. In our study, we restricted our designs to a limited number of simultaneous measurements of three commonly measured viral molecules, specifically the positive and negative sense RNA levels and the viral titre. Once again, PARSEC designs demonstrated significant improvements over random or anti-PARSEC designs (see Supplementary Figs. S17 and S18). This indicates that PARSEC is effective across various dynamical systems and has broader applicability.

We compared the distribution of the selected measurement time points between $\hbox {PD}_{{BC}}$ and $\hbox {RD}_{{BC}}$ to check for bias in selecting the measurements in PARSEC designs. Although there were no significant differences in the selection of the measurements in $\hbox {PD}_{{BC}}$ compared to $\hbox {RD}_{{BC}}$ (Fig. 4b), the average estimation error for $\hbox {PD}_{{BC}}$ was approximately three times lower than for $\hbox {RD}_{{BC}}$ (Fig. 4a). The clustering-based algorithm selects from a limited yet informative subset of the experiment design space, as demonstrated by the low variance in the estimation error (Fig. 4a) and the performance of sub-samplings of $\hbox {PD}_{{BC}}$ compared to $\hbox {RD}_{{BC}}$ (Fig. 4c).

We also observe that the performance of experiment designs generated by PARSEC(k) depends on how closely certain design specifications are implemented. For example, $\hbox {PD}_{{BC}}$ optimised the time points for the measurement of proteins B and C based on the corresponding PSI vectors. However, if we quantify proteins A and B at these optimised measurement time points ($\hbox {PD}_{{AB}}$), the estimation error increases compared to $\hbox {PD}_{{BC}}$. Nevertheless, $\hbox {PD}_{{AB}}$ still outperforms the random design quantifying proteins A and B ($\hbox {RD}_{{AB}}$) on average, with a 1.5 times lower error. The improved performance of $\hbox {PD}_{{AB}}$ compared to $\hbox {RD}_{{AB}}$ can be attributed to two factors: (i) $\hbox {PD}_{{BC}}$ accounts for the sensitivity profiles of protein B, which is a shared variable measured in $\hbox {PD}_{{AB}}$, and (ii) there may be a correlation between the sensitivity profiles of proteins A and C due to their coupled dynamics.

### PARSEC(k) tolerates parameter uncertainty

**Fig. 5:**
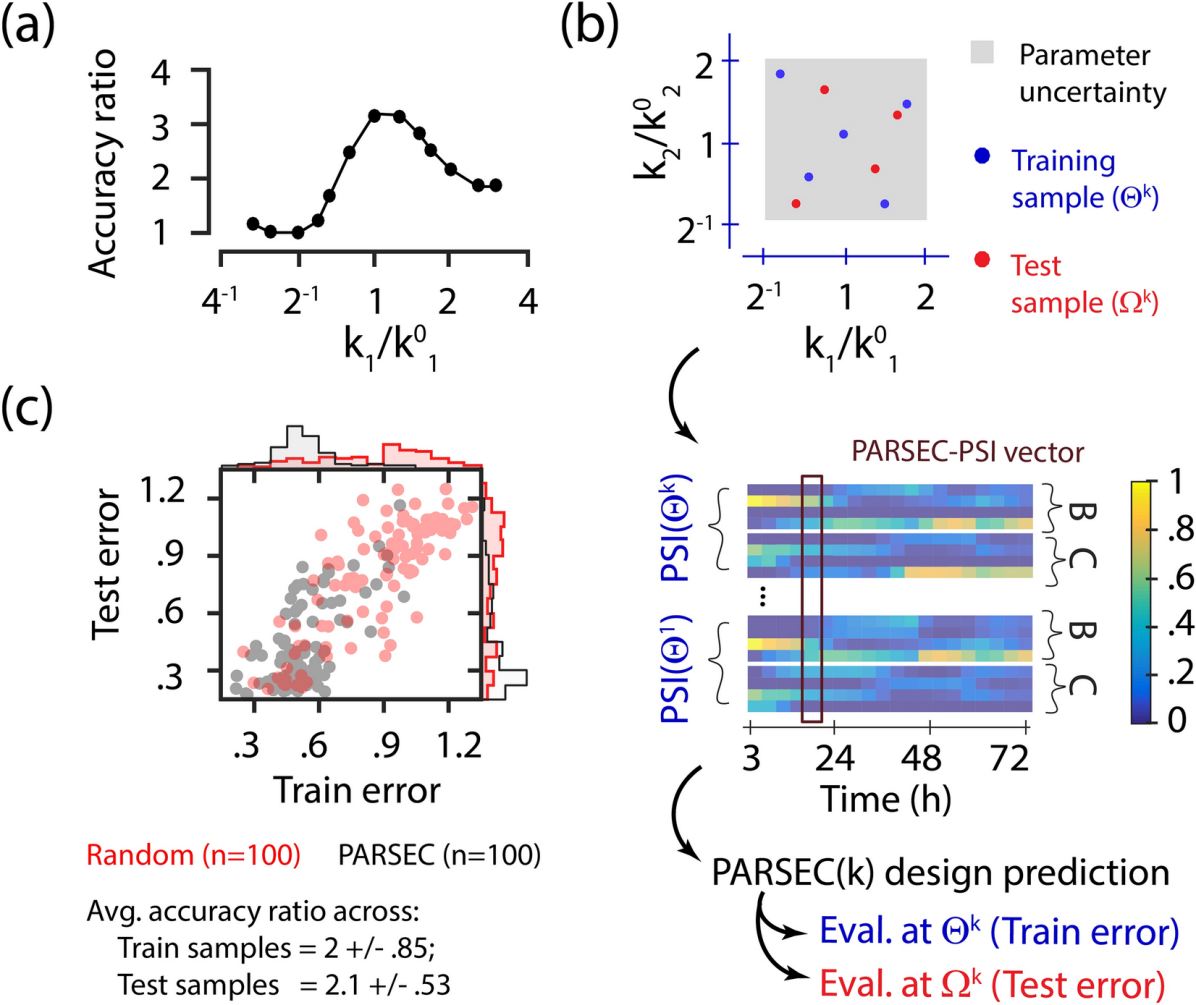
Robustness of PARSEC(k) designs. (**a**) Accuracy ratio evaluates how informative PARSEC(k) designs are compared to random designs. When the ground truth guess ($k_1^0$) is close to the actual ground truth ($k_1$), PARSEC(k) designs lead to about 3.2 fold lower estimation error compared to random designs. This accuracy ratio lessens as $k_1$ diverges from $k_1^0$, yet remains above 1, indicating superior designs by PARSEC. (**b**) A four-fold uncertainty characterised by a uniform distribution in log scale is considered in values of two of the six model parameters (see “Methods”). Using LHS, we identify five training parameter samples to inform the PARSEC-PSI vectors and predict the experiment designs. In addition to the training samples, four additional parameter combinations sampled via LHS (test samples, $\Omega ^k$), are also considered for design evaluation. (**c**) The scatter plot and the corresponding histograms shows the averaged estimation errors for each of the 100 PARSEC(k) (black scatter) and random (red scatter) designs evaluated at the train and test cases. The plot highlights that PARSEC(k) designs are more informative (i.e. have lower errors). The accuracy ratio is evaluated at each of the train and test samples, and their averages are reported in the bottom.

In the previous analysis, we optimised $\hbox {PD}_{{BC}}$ using PSI evaluated with a guess of parameter values ($\hbox {GT}_G$). However, the parameter guess is likely to be different from the true parameter value ($\hbox {GT}_T$). We evaluated PARSEC(k)’s ability to handle the discrepancy between the guess and the real value using the accuracy ratio. Here, the accuracy ratio is defined as the ratio of the mean estimation error for random design to that for PARSEC(k) designs. Our results indicate that although the accuracy ratio decreases as the initial guess diverges further from the ground truth, the PARSEC designs consistently exhibit higher accuracy (accuracy ratio > 1) across all parameter guess values within a four-fold range (Fig. 5a).

This inspired us to shift from relying on a single estimate to guide experiment designs to leveraging multiple initial parameter estimates, allowing PARSEC to account for uncertainty in parameter knowledge (Fig. 5b). To do this, PARSEC samples the associated distribution to generate representative guesses, referred to as training samples ($\Theta ^k$). The PSI values evaluated at the training samples are then combined to form PARSEC-PSI vectors, which are used for clustering and experiment design selection. The estimation errors for PARSEC (k) and random designs are evaluated using data generated at the training samples ($\Theta ^k$) and validated in another set of parameter samples (test samples, denoted as $\Omega ^k$).

Our analysis shows that for the three gene repressilator model, the average of the accuracy ratios over the training and test samples are 2 ± .85 and 2.1 ± .53, respectively (Fig. 5c). This suggests that PARSEC(k) designs are consistently twice as informative than the random designs at any parameter combination. Furthermore, the top 5% of PARSEC(k) designs in the training set also perform well in the test cases inspite of their differences in parameter values. This indicates that these experiment designs are resilient to parameter uncertainty, making them suitable for universal designs for the model system. As an additional support, we show that PARSEC outperforms the random designs even when the uncertainty in parameter values is larger (Supplementary Fig. S12), i.e., nine-fold uncertainty compared to four-fold considered here.

### Optimising the sample size

**Fig. 6:**
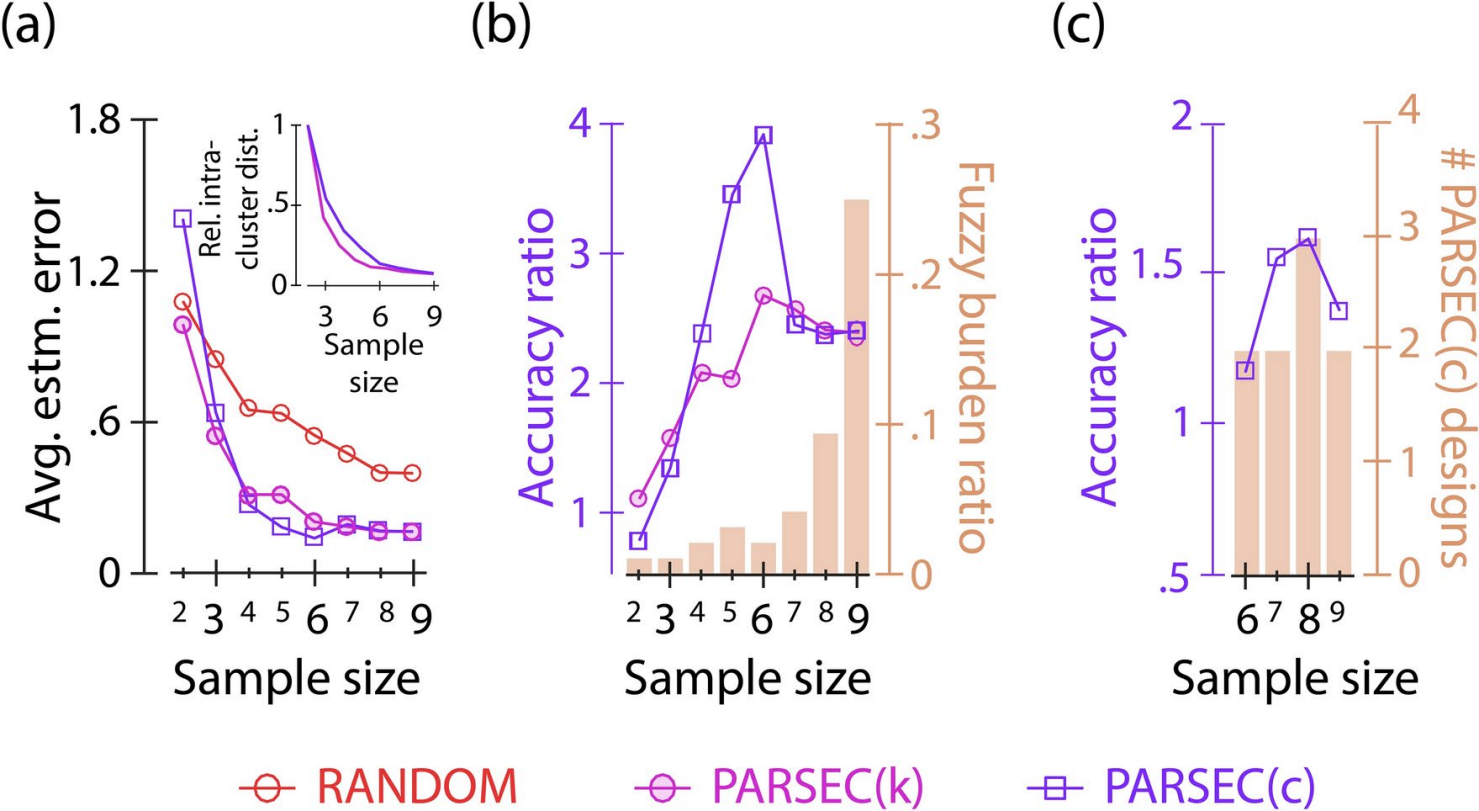
Optimising experimental sample size. (**a**) Average estimated error for PARSEC(k), PARSEC(c), and random designs of the repressilator model as a function of the sample size is shown. The inset shows the variation of the intra-cluster distance as a function of number of clusters considered during k-means (open circle) and c-means (open square) clustering employed by PARSEC(k) and PARSEC(c), respectively. (**b**) Line plot of the accuracy ratio of PARSEC(k) (open circle) and PARSEC(c) (open square) is shown. The fuzzy burden ratio that reports the ratio of distinct PARSEC(c) designs and PARSEC(k) designs evaluated is shown as bar plots. (**c**) The accuracy ratio (line plot) of predicted PARSEC(c) designs as a function of the sample size is shown for the repressilator model analysis. The bar plot shows the number of unique experiment designs obtained while considering 100 realisations of PARSEC(c), as a function of sample size. 100 realisations each of random and PARSEC(k) were considered for each sample size.

We then investigated whether PARSEC could help us determine the optimal sample size for the experiments. As anticipated, increasing the sample size in both random and PARSEC(k) designs leads to more precise parameter estimation (Fig. 6a). However, the marginal gain in estimation accuracy decreases steadily with sample size (Supplementary Fig. S13). Results indicate that PARSEC(k) designs for the repressilator model with six or more samples yield comparable outcomes on average (Fig. 6a), suggesting that an experiment design with six measurements would be economical. Notably, the intra-cluster distance observed during clustering also hits an inflection point at approximately six clusters, suggesting that the ideal partitioning of the PARSEC-PSI clusters occurs with the same number of sampling points (Fig. 6a-inset).

Based on this we propose that the PARSEC-PSI vector clustering efficiency can be a valuable metric for determining the sample size for PARSEC, potentially resulting in high accuracy ratios. This can be explained by two opposing factors. First, the use of too few clusters restricts the representation of PARSEC-PSI vectors in PARSEC designs. Therefore, increasing the number of clusters increases the representation and information content of the experiment designs, thus improving the accuracy ratio. Conversely, an excessive amount of clustering results in an overabundance of similar (or identical) PARSEC-PSI vectors, which only provides a minor increase in the information content when compared to randomly generated designs. Thus, PARSEC prevents over-sampling by identifying an optimal sample size. The observed correlation between clustering efficiency and accuracy ratio further suggests the effectiveness of the PSI-clustering-based approach in experiment design.

Our version of PARSEC(k) reliably estimates the optimal sample size, albeit with significant computational cost due to the need to assess various designs. This cost increases further when taking into account the uncertainty of parameters across different values. The requirement for numerous predictions generated in PARSEC(k) stems from the randomness involved in discretely grouping continuous PARSEC-PSI vectors via the k-means clustering approach. An alternative is to use the fuzzy c-means clustering (Supplementary Fig. S14) as implemented in PARSEC(c). This method reduces the number of unique optima, significantly decreasing the computing cost (Fig. 6b—bar plot). PARSEC(c) shows similar cluster convergence (Fig. 6a: inset) and comparable estimation error and accuracy ratio profiles (Fig. 6a,b) when changing sample sizes while requiring almost twenty times fewer computations (Fig. 6b—bar plot).

This improved efficiency makes it possible to optimise the sample size for a generalist experiment design search using PARSEC(c). Generalising the analysis in Fig. 5c, we identified generalist designs for various sample sizes using PARSEC(c). PARSEC(c) designs with eight samples show the highest accuracy ratio (Fig. 6c, Supplementary Figs. S15 and S16). Nevertheless, this is established with significantly fewer computations in PARSEC(c) now. For example, we require only two PARSEC(c) designs compared to one hundred previously used PARSEC(k) designs to report efficient designs involving experiments with six time points (see Fig. 5c).

## Methods

### Implementation of ABC-FAR

The ABC-FAR method iteratively selects parameter combinations and either rejects or accepts them based on the associated $\chi ^2$ values, which measure the difference between the data ($R_{D}$) and the corresponding model prediction ($R_{P}$). We utilise the $\chi ^2$ statistics to enhance the goodness-of-fit as proposed earlier^34^. The $\chi ^2$ is determined as: $\chi ^2 = \sum \frac{(R_{D} - R_{P})^2}{\sigma _{D}^2}$, where the sum is taken over all data points and $\sigma _{D}$ represents the standard deviation in data $R_{D}$.

In each iteration, a noisy version of the current estimate of the distribution is sampled. The magnitude of the added noise is reduced with each iteration. Let $p^{(k-1)}_{\theta _j}$ be the current estimate of the marginal of the parameter $\theta _j$ at the $k^{th}$ iteration. The noisy distribution ($m^k_{\theta _j}$) is then created using a uniform distribution ($U_{\theta _j}$) in the following way:

$$\begin{aligned} m^k_{\theta _j} = \frac{0.25}{k+1} \times U_{\theta _j} + \left( 1 - \frac{0.25}{k+1}\right) \times p^{(k-1)}_{\theta _j}. \end{aligned}$$

where for k > 1, $p^{(k-1)}_{\theta _j}$ is the posterior obtained from the (k-1)$^{th}$ iteration and $p^{0}_{\theta _j}$ denotes the prior or initial guess. The support of the uniform distribution matches the bounds of the prior distribution, ensuring that parameter estimates remain within these bounds.

We use Latin Hypercube Sampling (LHS^35^) for sampling each parameter independently from its marginal. Although this approach improves robustness, it might slow down convergence. Gradient noisy sampling, as discussed above, aids in exploring the parameter space during early iterations without significantly hindering the convergence in later iterations.

In every iteration, we apply a history-dependent update strategy (HDUS) that recalls all the parameter combinations selected in the prior iteration while comparing the parameter combinations sampled in the current sampling step. This aids in preserving the joint distribution to a certain degree, ensuring that advantageous parameter combinations are not overlooked, and securing a monotonic reduction in the $\chi ^2$ statistic. Alternatively, we can use a history-independent update strategy (HIUS), which does not retain previously chosen combinations (see Supplementary Figs. S6 and S7). Although HIUS conserves computational memory, it does not guarantee convergence under weak rejection conditions (high FAR values).

To identify computational artifacts and evaluate statistical significance, we apply and compare the ABC-FAR results of a dummy parameter. In addition, we evaluate the Pearson’s correlation coefficient (PRCC, Supplementary SI S5) and linear regression between pairs of chosen parameter combinations. The PRCC estimates practical non-identifiability, while the regression indicates the expected parameter adjustment needed to counterbalance changes in the $\chi ^2$ function due to fluctuation in the corresponding partner parameter value. These properties are linear and local, depending on the values of the other model parameters.

### Implementation of PARSEC

#### Measurement candidates and parameter sampling

Let ${\mathcal {M}} = \{M_1, M_2, \ldots , M_n\}$ represent the set of all feasible measurement candidates, selected based on experiment design specifications. Further, let ${\mathcal {V}}$ denote the parameter space defined by the known uncertainty. A combination of parameters is a p-component vector, $\Theta = [\theta _1, \theta _2, \ldots , \theta _p]^\text {T} \ \in {\mathcal {V}}$, where $\theta _i$ denote the value of the $i^\text {th}$ parameter of the model. Here again, we use Latin Hypercube Sampling (LHS) to sample this space to generate a set of training samples ${\mathcal {S}} = \{\Theta _1, \Theta _2, \ldots , \Theta _k\}$, where $\Theta _i$ is a sampled parameter vector.

#### Parameter sensitivity analysis

For each candidate measurement $M_i \in {\mathcal {M}}$ and each sample $\Theta _k \in {\mathcal {S}}$, we calculate the Parameter Sensitivity Index (PSI) vector using the extended Fourier Amplitude Sensitivity Test (eFAST)^38,39^. Briefly, eFAST is a global sensitivity analysis method that uses variance decomposition to assess the influence of input parameters on model outputs. It evaluates sensitivity index as the ratio of expected variance in output variable in two conditions: (a) when only the parameter of interest changes, and (b) when all model parameters vary (more details in^38,39^).

Let $\psi (M_i, \Theta _j, \theta _i)$ denote the PSI of candidate $M_i$ due to fluctuations in the values of the $i^\text {th}$ parameter, $\theta _i$, evaluated at training sample $\Theta _j$. The corresponding PSI vector for $M_i$ at $\Theta _j$, $\Psi (M_i, \Theta _j)$, is given by

$$\Psi (M_i, \Theta _j) = [\psi (M_i, \Theta _j, \theta _1), \psi (M_i, \Theta _j, \theta _2), \ldots , \psi (M_i, \Theta _j, \theta _p)]^\text {T}$$

All of these vectors are combined to form the PARSEC-PSI vector,

$$\text {PARSEC-PSI}(M_i) = [\Psi (M_i, \Theta _j), \Psi (M_i, \Theta _j), \ldots , \Psi (M_i, \Theta _k)]^\text {T}$$

#### Clustering and selection of the measurement candidates

We next apply a clustering algorithm (either k-means or fuzzy c-means) to the set of PARSEC-PSI vectors. We consider the Euclidean distance between two PARSEC-PSI vectors for clustering the measurement candidates into a pre-defined number of clusters, represented by $C_1, C_2, \ldots , C_k$. The clustering criterion aims to minimise intra-cluster distances, formally given by

$$\begin{aligned} \min \sum _{i=1}^{k} \left\{ \sum _{\text {PARSEC-PSI}(M_i) \in C_i} \text {distance}(\text {PARSEC-PSI}(M_i), \mu _{C_i}) \right\} \end{aligned}$$

where $\mu _{C_i}$ is the centroid of cluster $C_i$. For PARSEC(c) using fuzzy c-means, we select measurement candidates based on the highest membership probability from each cluster. For PARSEC(k) using k-means, a candidate is randomly selected from each cluster.

#### Design evaluation and parameter estimation

We evaluate designs at different parameter values $\Theta = [\theta _1, \theta _2, \ldots , \theta _p]^T \in {\mathcal {V}}$ by simulating the model. Let $D(G, \Theta )$ denote the dataset generated emulating design *G* at $\Theta$. We employ ABC-FAR to estimate parameters using $D(G, \Theta )$. Let ${\hat{\theta }}^m_j$ denote value of the $j^{th}$ parameter of the $m^{th}$ parameter combination selected in the last iteration of ABC-FAR. We calculate the estimation error as:

$$\begin{aligned} \text {Estimation error} = \sqrt{ \sum _{j = 1}^{r} \left\{ \sum _{m = 1}^{M} (\theta _j - {\hat{\theta }}^m_j)^2 \right\} }. \end{aligned}$$

*r* and *M* indicate the number of free model parameters to be estimated and the number of parameter combinations selected by ABC-FAR, respectively.

For PARSEC, we employ ABC-FAR to refine the posterior five times, beginning with a prior uniform distribution on a logarithmic scale that spans two orders of magnitude. We use the history-dependent update strategy and a FAR value of 0.01 (unless otherwise specified). In each iteration, we sample 60,000 parameter combinations using a noisy version of the current estimate of the marginal (as described in the preceding section). More details on PARSEC implementation and design evaluations are provided in the supplementary SI S1.

### DoE for a three-gene repressilator system using PARSEC

We investigate the behavior of a three-gene repressilator system, a synthetic genetic circuit defined by the cyclic inhibition of gene expression^37^. By employing an ordinary differential equation (ODE) model as described in Supplementary SI S3, we track the concentrations of the proteins produced by these genes.

To mirror the constraints of the experiment, only measurements of protein B and C are permitted. Furthermore, to emulate real-world conditions, the entire duration of the experiment is limited to 72 hours, and measurements of both proteins must be taken simultaneously. As an example, we design and compare sets of six concurrent measurements of protein B and C levels over the 72 hours to determine parameter values using PARSEC and its alternatives. The algorithm pertinent to the design issue is explained below (refer to Supplementary Fig. S1 b).

1. To begin with, we need to create a set of all potential measurement candidates. Apart from the design criteria, we restrict the time points to multiples of three hours to discretise the experiment’s time axis and limit the computations. Thus, six elements must be chosen from the set $T_F = \{3, 6, 9, \ldots , 69, 72 \}$ hours to enhance parameter estimation accuracy. These selected time points, model equations, and variables of interest will be inputs into the algorithm. The problem specifies the variables of interest as the levels of proteins B and C.
2. We then compute the parameter sensitivity indices for each variable of interest at each viable time point. This sensitivity analysis uses a nominal guess of the combination of parameter values. Since we need to measure the levels of proteins B and C simultaneously, we combine the parameter sensitivity profiles of each variable to create the concatenated PARSEC-PSI vector for each feasible measurement time point.
3. Next, the measurement candidates (time points for measurement), are grouped into six clusters (corresponding to the six measurement time points) based on similarities in their PARSEC-PSI vector. We apply the k-means clustering algorithm on the Euclidean distance between the PARSEC-PSI vectors. Since k-means clustering is stochastic in nature, it can produce varying cluster groupings in different iterations, we generate design and compare results from several clustering runs.
4. For each iteration, one measurement candidate is picked randomly from each of the six clusters to define the final designs considered for evaluation by ABC-FAR for parameter estimation ($\hbox {PD}_{{BC}}$).

We generate one hundred PARSEC designs and evaluate them against random designs ($\hbox {RD}_{{BC}}$ and $\hbox {RD}_{{AB}}$), non-specific designs ($\hbox {PD}_{{AB}}$), and anti-PARSEC designs ($\hbox {WD}_{{BC}}$) using six concurrent measurements of the two variables. For each mechanism, we produce a hundred designs, except for $\hbox {WD}_{{BC}}$, where only 40 designs are generated.

To assess the robustness of PARSEC configurations in the face of parameter uncertainty, we employ a uniform distribution (on a logarithmic scale) covering a four-fold range of parameter values for two out of the six parameters in the repressilator model (Figs. 5b and 6c). The PARSEC-PSI vector is constructed using PSI calculated at five training values ($\Theta ^k$), which are chosen based on Latin Hypercube Sampling (LHS) from the uncertainty range. The experimental design is then evaluated using these five training parameter sets and an additional set of four test parameter sets ($\Omega ^k$), also selected through LHS. Given the breadth of uncertainties considered, we extend the duration of the experiment measurements to 120 hours in this analysis.

To determine the optimal sample size (Fig. 6a, b), we produce the PARSEC-PSI vectors with a fixed set of parameter values for 72 hours. The sample size defines the number of groups for clustering the PARSEC-PSI vectors. To score the performance of PARSEC and identify a good sample size, we calculate the accuracy ratio, as the ratio of averaged estimation error for random designs to that for PARSEC designs, analysed for a given experiment design specifications. Thus higher the accuracy ratio, the more advantageous the use of PARSEC. We also define the ‘fuzzy burden ratio’ to mark the (inverse of the) computational efficiency achieved through the use of fuzzy c-means clustering as opposed to k-means clustering. The burden ratio looks at the ratio of the number of distinct designs evaluated under the PARSEC(c) framework to that under the PARSEC(k) framework. A lower burden ratio suggests higher efficiency.

### DoE for a viral life cycle model using PARSEC

Our PARSEC experiment designs for the life cycle of a positive-sense RNA virus are based on a recently published model^32^. This model outlines the cellular growth dynamics of various viral molecules, including different viral RNA species, viral replication intermediates, viral proteins, and the virus particles that are released from the cell. It incorporates eleven parameters to describe the interactions between these virus growth variables.

Seven of these parameters (mentioned under ‘Free parameters’ in Supplementary Table S2) are directly associated with the viral life cycle stages of translation, replication, and packaging and are expected to change with the virus strain. Therefore, for implementation of PARSEC to this model, we devise experiments aimed at estimating these seven parameters. We tailor the experimental designs to include only concurrent measurements of three commonly quantified viral molecules: positive and negative sense RNA levels and viral titre. In addition, we set a fixed sample size and restrict the experiment duration to 48 hours. We discretised the experiment time axis in intervals of two hours. As a result, PARSEC selects a fixed number of time points from the set: $T_F = \{2, 4, 6, \ldots , 46, 48 \}$ Further, we consider predetermined initial conditions and prior knowledge of the parameters (details in Supplementary Tables S2 and S3).

## Discussion

The PARameter SEnsitivity Clustering-based algorithm (PARSEC) leverages the structure of the model through parameter sensitivity to optimise experimental design. Our findings with PARSEC(k) show that the spread of measurement candidates resembles that of random designs (Fig. 4b). However, the estimation errors from PARSEC-derived measurements are significantly lower and more tightly contained (Fig. 4a). This suggests that the overlap (or similarity) between PARSEC-PSI vectors plays a more pivotal role in DoE than the distinct characteristics of the individual measurements in the PARSEC sets. At present, we randomly select candidates from each cluster, but a more systematic selection could further enhance the effectiveness of the experiment design exploration. The parameter estimation error decreases with a reduction in the overlap of the parameter sensitivity (PSI) vectors of measurements (comparison of $\hbox {PD}_{{BC}}$ and $\hbox {WD}_{{BC}}$, Fig. 4a) highlighting the advantage of our PARSEC approach. Finally, we demonstrate that the effectiveness of PSI clustering is correlated to the accuracy ratio of the designs as the sample size varies (Fig. 5d). Hence, the benefit of clustering parameter sensitivity profiles during the design generation process is evident, and this study presents a framework to determine an appropriate estimate of the optimal sample size for the experiment.

The key components of the algorithm are the PARSEC-PSI vectors. Our strategy of vectorially conjoining the PSIs to form the PARSEC-PSI vectors allows us to identify experiment designs that are resilient to parameter uncertainty as well. We join the PARSEC-PSI evaluated at different parameter combinations (sampling the uncertainty). This creates the high-dimensional PARSEC-PSI vectors used for subsequent clustering and design selection. Our results clearly show that even with uncertainty in parameter values, PARSEC outperforms the random designs. The resilience of these designs to parameter uncertainty is an attractive characteristic that distinguishes PARSEC from locally optimal MBDoE techniques like those based on Fisher’s Information Matrix. Such robustness to parameter uncertainty is accompanied by a decrease in the accuracy of parameter estimation obtained from the experiment designs. Therefore, depending on the level of uncertainty, the researcher can use PARSEC to either plan a ‘robust generalist’ design or a ‘locally optimised’ design.

The PSI vector-conjunction approach enables us to easily incorporate various measurement condition constraints as well. For example, in our current analysis, we enforced simultaneous measurements of the different variables. Alternatively, if measurements of different variables have to be taken at a defined time offset from each other, then the PSI of the variables can be combined in a staggered fashion to generate the final PARSEC-PSI vectors. In the absence of any restrictions, individual variables can be treated as separate measurement candidates for clustering and experiment design selection (see Supplementary SI S1 and Supplementary Fig. S1). Moreover, the type of measurement also affects design specifications. For example, certain (variable) measurements may be more cost-effective or easier to perform than others. In those cases, PARSEC can be extended to identify variable combinations for economical and informative designs. Similarly, other specifications such as initial conditions, can also be adjusted to improve designs for parameter estimation.

The effectiveness of each experiment design is scored on the basis of the deviation of these estimated values from the known ground truth, allowing us to identify and select the most efficient and yet informative designs. PARSEC utilises the Approximate Bayesian Computation-Fixed Acceptance Rate (ABC-FAR) algorithm to automate and equitably compare experimental designs. The ABC-FAR takes into account a global and relative sampling-rejection criterion (FAR), which circumvents setting a data-dependent error threshold and reduces the need for trial and error, ensuring robust convergence. Moreover, a global parameter sampling technique that includes simulated annealing is employed to enhance the robustness of estimates and prevent local minima and biases. We have validated the algorithm for accuracy, efficiency, and robustness to initial bias^32^ and measurement noise. ABC-FAR is also amenable to incorporation of available system-specific knowledge to improve convergence accuracy and speed (Supplementary Fig. S10). Furthermore, we are able to identify data-specific correlations and dependencies among the estimated parameter values, which can be used to infer model features, such as practical identifiability. ABC-FAR can be modified in future work to facilitate model selection by considering the model identity as a variable parameter. ABC-FAR parameter estimation efficiency can be further enhanced by iteratively adjusting the FAR value based on convergence improvements. Additionally, like other ABC-based methods, ABC-FAR can be extended to estimate parameter values under more complex measurement noise^40,41^ and to stochastic dynamical systems^42^. In summary, ABC-FAR proves to be a highly effective parameter estimation method with broad applicability that extends well beyond its essential function within PARSEC.

PARSEC employs the clustering of combined parameter sensitivity vectors to evaluate and select experimental designs. We utilise k-means clustering, a widely-used clustering technique, to create the PARSEC-PSI clusters (PARSEC(k)). An alternative approach to minimise computational costs is fuzzy clustering as employed in PARSEC(c). It is essential to highlight that PARSEC(c) designs, which use fuzzy clustering, are a subset of PARSEC(k) designs. Although PARSEC(c) is effective in identifying good designs quickly, it can overlook the most informative ones. In contrast, PARSEC(k) performs a more comprehensive search to pinpoint the most informative designs.

Although we employ popular techniques from the field, the choice of sensitivity analysis and clustering tools in the PARSEC framework is highly adaptable. Users have the flexibility to choose tools according to their particular assumptions, relevance, and standard methodologies in their discipline, enabling tailored and enhanced algorithm performance. Nonetheless, the fundamental assumptions of the clustering and sensitivity analysis tools utilised can also restrict the functionality and applicability of PARSEC, necessitating thoughtful selection. Additionally, in the current implementation, PARSEC assumes a discretised set of design variables (measurement candidates) and hence it is sensitive to design variable discretisation as well. The last step of PARSEC is computationally demanding, similar to other Bayesian Model-Based Design of Experiments (MBDoEs). However, the tight distribution of low estimation errors in PARSEC(k) implies that the PARSEC experiment designs are usually informative (Fig. 4a). Thus, a smaller set of PARSEC(k) predicted designs (Fig. 4c) can be sufficient to identify a good experiment design.

In summary, PARSEC uses parameter sensitivity analysis to infer the information content of the measurables and performs clustering to limit redundancy in the information collected in an experiment. This enables the identification of informative and generalist experiment designs of optimal sample size, which is a precursor for effective experiments.

## Supplementary Information

Supplementary Information.

## Data Availability

The data used in the study were computationally generated. The codes for data generation and analysis are available for download from our Github repository, https://github.com/hcharsh/PARSEC_ABC_FAR. A version of the code compatible with SBML is also uploaded there.

## Declarations

### Declaration of AI use

We used AI-assisted technologies in the editing of the draft of the article for clarity and readability. No other AI tools were used in the creation of the article.
