# Supplementary material for: Rationalised experiment design for parameter estimation with sensitivity clustering
Source: Sci Rep. 2024 Oct 28;14:25864. doi: 10.1038/s41598-024-75539-2 (PMC11519581; doi:10.1038/s41598-024-75539-2)
Supplement: Supplementary file 1 — Supplementary Information. [file 41598_2024_75539_MOESM1_ESM.pdf]

Rationalised experiment design for parameter estimation with sensitivity clustering

Harsh Chhajer[a], Rahul Roy[a,b]*

Author Affiliations:

[a] Department of Bioengineering Indian Institute of Science, Bangalore 560012, India.

[b] Department of Chemical Engineering, Indian Institute of Science, Bangalore 560012, India.

Orchid identifiers: HC: 0000-0001-9252-1855; RR: 0000-0003-3329-8803

*Corresponding author: Rahul Roy.

Department of Chemical Engineering, Indian Institute of Science, Bangalore, Karnataka, India-560012.

Phone: 91-80-2293-3115 E-mail: rahulroy@iisc.ac.in

Article classification: Design of experiment | Computational biology

Keywords: Approximate Bayesian computation | Model fitting | Informative experiment design | Parameter sensitivity | Clustering-based experiment design.

Supplementary Information

Rationalised experiment design for parameter estimation with sensitivity clustering

Harsh Chhajer, Rahul Roy

SI S1 Constructing the PARSEC-PSI vector

The following were introduced in the main text:

- $\mathcal{M} = \{M_1, M_2, \ldots, M_n\}$ represent the set of all feasible measurement candidates (M_i), selected based on design specifications, like time span of experiment, possibility of simultaneous measurements, and so on.
- $\mathcal{V}$ denotes the parameter space defined by the existing knowledge of the parameter values prior to the experiment.
- $\Theta = [\theta_1, \theta_2, \ldots, \theta_p]^{\mathrm{T}} \in \mathcal{V}$ denotes a realization of parameter combination in the given uncertainty.
- $\psi(M_i, \Theta_j, \theta_i)$ denotes the PSI of candidate M_i, or how sensitive the value of M_i is to the fluctuations in the values of the ith parameter, θ_i, evaluated at training sample Θ_j
- $\Psi(M_i, \Theta_j) = [\psi(M_i, \Theta_j, \theta_1), \psi(M_i, \Theta_j, \theta_2), \ldots, \psi(M_i, \Theta_j, \theta_p)]^{\mathrm{T}}$ is PSI vector for M_i evaluated at Θ_j
- PARSEC-PSI(M_i) $= [\Psi(M_i, \Theta_j), \Psi(M_i, \Theta_j), \ldots, \Psi(M_i, \Theta_k)]^{\mathrm{T}}$ is the PARSEC-PSI vector corresponding to M_i which will be used for clustering.

In the methods section of the main text, we just focused on the case of a simple measurement candidate - the measurement comprised of a scalar quantifying just one variable at a particular time point. The PSI vector for the scalar M_i is well-defined. However in most systems, a synchronous observation of multiple variables ($V_1, V_2, \ldots V_q$) is involved. This renders M_i to be a vector of variables, necessitating a revised definition of PSI vector of M_i. Suppose the synchronicity of measurement constrains that the variable V_i must be measured at a time point that is offset by Δ_i time units after the measurement of variable V_1. By definition $\Delta_1 = 0$; and the special case of simultaneous measurement of variables would imply zero time offset, that is, $\Delta_2 = \Delta_3 = \ldots = \Delta_q = 0$.

Here we demonstrate how to construct the PARSEC-PSI vector for measurements involving a synchronous observation of q variables (Supplementary Figure S1).

Let M_i denote the measurement candidate whose V_1 is measured at time t_i. Thus $M_i = [V_1(t_i), V_2(t_i + \Delta_2), \ldots, V_q(t_i + \Delta_q)]^T$ is a vector made of scalar quantities $V_k(t_i + \Delta_k)$. PSI vectors of scalar like $V_k(t_i + \Delta_k)$ is well-defined. So $\Psi(V_k(t_i + \Delta_k), \Theta_j)$ is the PSI vector of $V_k(t_i + \Delta_k)$ evaluated at Θ_j. We

define the PSI vector of M_i by vectorially joining the PSI vectors of the individual variables, $V_k(t_i + \Delta_k)$, together.

$$\Psi(M_i, \Theta_j) = [\psi(V_1(t_i), \Theta_j), \psi(V_2(t_i + \Delta_2), \Theta_j), \ldots, \psi(V_q(t_i + \Delta_q), \Theta_j)]^{\mathrm{T}}$$

$\Psi(M_i, \Theta_j)$ turns out to be a $(q \times r)$-dimensional vector. These PSI vectors evaluated at each of the u training sample of parameter combinations (Θ_u) are again conjoined to give the PARSEC-PSI vector for each measurement.

$$\text{PARSEC-PSI}(M_i) = [\Psi(M_i, \Theta_1), \Psi(M_i, \Theta_2), \ldots, \Psi(M_i, \Theta_u)]^{\mathrm{T}}$$

which is a $(u \times q \times r)$-dimensional vector.

The last equation is same as in the main text. PARSEC-PSI(M_i) turns out to be $(q \times r)$-dimensional vector.

SI S2 ABC-FAR algorithm for model parameter estimation

We propose a likelihood-free, Approximate Bayesian computation- (ABC) based algorithm for fitting the model and estimating corresponding parameter values (Figure 2a, Main text). We use Monte-Carlo sampling technique to avoid explicitly calculating the (typically intractable) likelihood as a function of the parameter value. Instead, we calculate the deviation between model prediction and data (denoted as χ^2), by simulating the model for sampled parameter combinations. We select a fixed fraction of the number of the sampled parameter combinations with the lowest χ^2 value to update the current distribution. We denote the value of this fixed fraction as the Fixed Acceptance Rate or FAR. The process of sampling from the current distribution, selection of parameter combination and updating the current distribution, is repeated to narrow down the plausible parameter value range. This concentrates computations in the later iterations to narrower ranges where the parameter values are more likely to lie, making the algorithm efficient. In our analysis, we include an additional dummy parameter that doesn't affect model prediction but undergoes the same algorithmic treatment as the model parameters being estimated. It is used to (a) detect artifacts, if any introduced by the algorithm, and (b) inform on significance of properties of estimated model parameters.

Here we describe the parameter sampling and acceptance modules of the algorithm. Suppose the model being fit, has k parameters. In each iteration, N realizations of parameter combination are sampled using the current estimate of the marginal of the k model parameters. For the first iteration, the current estimate of marginal would be the prior distribution (initial guess), whereas in subsequent iterations, the current estimate is the posterior estimated in the previous iteration. The algorithm employs Latin Hyper-Cube Sampling (LHS, [1]) to effectively scan the (potentially high-dimensional) parameter space. A noise, whose magnitude decreases with iteration, is added to the distribution each time before sampling. This helps in exploring the parameter space better in the earlier iterations without hindering the convergence,

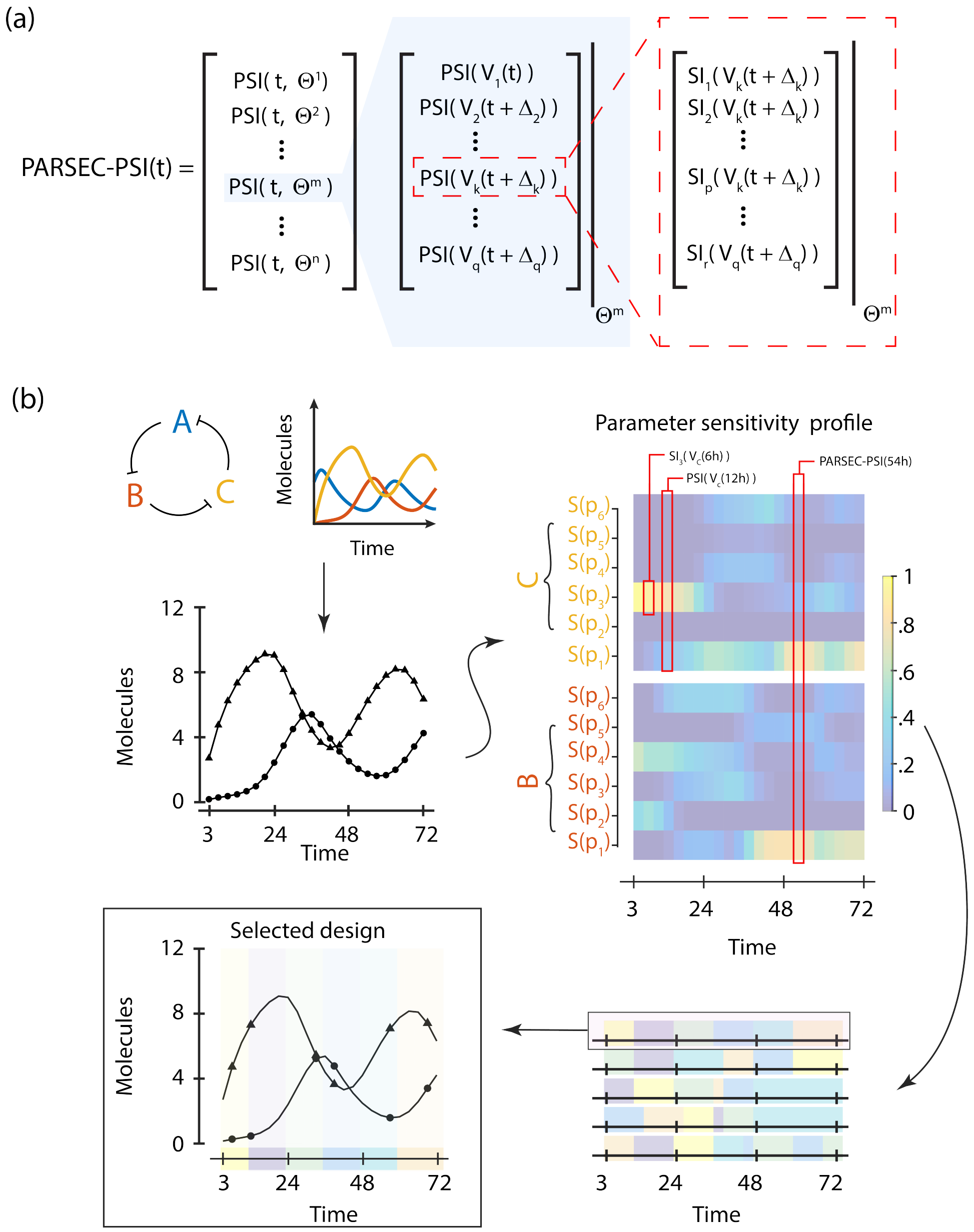

Figure S1: **Constructing the PARSEC-PSI vector and Implementing PARSEC for the repressilator system** (a) PARSEC relies on the clustering of the PARSEC-PSI vectors. Thus these vectors are the main functional components of the PARSEC algorithm. PARSEC allows us to design these vectors to account for parameter uncertainty and various experiment design constraints. For example, suppose we want to characterize a kinetic system by measuring q variables synchronously. We show the procedure for constructing the PARSEC-PSI vectors designs consisting of measurement candidates (M_i) involving the synchronized measurement of q variables $(V_1, V_2, \ldots V_q)$. (b) To implement PARSEC for the repressilator system, we first identify the set of feasible measurement candidates (or measurement time points). We evaluate the parameter sensitivity profile for each candidate by concatenating the parameter sensitivity indices for levels of protein B and C for each of the six parameters. The candidates are clustered into six groups (dictated by defined sample size), and one time point of measurement is selected from each cluster to form the selected design.

significantly, in the later ones, like in simulated annealing [2]. Adding a sampling noise helps avoid local minima in the earlier iterations and improves robustness of the estimation.

The model is simulated for each of the N parameter combinations sampled. Appropriate model predictions are compared to the data, to estimate χ^2 statistics as a measure of the deviation. χ^2 is the weighted sum of the squared of difference between prediction and data, weighted by variance in measurement. Desirable model fits should closely match the data. We select the M (= N $\times$ FAR) parameter combinations corresponding to the lowest χ^2 (best agreement between prediction and data) to update the estimate of the distribution of the parameter values.

From the second iteration and onwards, we can choose to include previously selected parameter combinations, while sorting the N sampled combinations and updating the posterior in an iteration. This 'history-dependent update strategy' (HDUS) ensures that newly sampled parameter combinations are only accepted if they are better than those selected earlier. On the other hand, the 'history-independent update strategy' (HIUS) selects parameter combinations solely from the current sampling to update the posterior. Irrespective of the update strategy, in each iteration, N model parameter combinations are sampled and only M parameter combinations are selected. This maintains an acceptance rate of FAR = M/N, in each iteration. However the total number of sampling and thus computations increases every iteration. Thus the computation efficiency falls inversely with iteration and is characterized by the effective acceptance rate (EAR = $\frac{\mathrm{M}}{\mathrm{N}\times\text{iteration index}}$). In this article, we discuss how the choice of update strategy and FAR values affect efficiency and convergence of the algorithm, after we demonstrate the working of the algorithm.

Keeping the initial condition fixed, we employ the ABC-FAR algorithm, to fit the model to the data. We consider N = 10^4 samplings and a FAR (fixed acceptance rate) of 2.5% to update the posterior, which is iterated eight times. We use the . Thus second iteration and onwards, the algorithm selects 250 (M = N$\times$FAR = 250) best parameter combinations from a collection of 'N (sampled in the current iteration) + M (selected in the previous iteration)' combinations.

SI S3 Description of the systems considered

Prey-predator system (Lotka-Volterra model). We look at the popular Lotka Volterra model, which is typically used to benchmark parameter estimation algorithms (as in [3]). The model tries to explain the population dynamics due to the interaction between prey and predator species. In the model, the population of prey (N) increase exponentially in absence of the predators (associated parameter: a). If predators are present, they interact with and consume the prey. This leads to a decrease in the prey population (associated parameter: b), but promotes the proliferation of predators (associated parameter: c). However, in absence of preys, the predator population (P) decays exponentially (associated parameter: δ), due to lack of food/resources.

Lotka-Votka model

$$\frac{dN}{dt} = a.N - b.N.P \tag{1.1}$$

$$\frac{dP}{dt} = c.N.P - \delta.P \tag{1.2}$$

Three-gene repressilator system. The system consists of three genes regulating each other's expression. Expression of one gene produces a protein, that represses the expression of the other gene in cyclic manner.

GRN model

$$\frac{dA}{dt} = -\delta.A \; + r_{base} \; + \frac{k_1}{1 \; + \; (\frac{C}{k_4})^4} \tag{2.1}$$

$$\frac{dB}{dt} = -\delta.B \; + r_{base} \; + \frac{k_1}{1 \; + \; (\frac{A}{k_2})^4} \tag{2.2}$$

$$\frac{dC}{dt} = -\delta.C \; + r_{base} \; + \frac{k_1}{1 \; + \; (\frac{B}{k_3})^4} \tag{2.3}$$

Coherent feed forward (type-1) loop - FFL-1 We look at a coherent feed-forward (type-1) network for gene regulation. Molecules of species S (population size - S) activates the production of protein A (population size - A). Presence of A induces the degradation of S. Protein A activates the production of a protein B (population size - B). Presence of both the proteins, A and B, promotes the production of another protein C (population size - C). Additionally we also consider a natural decay/dilution kinetics for the proteins A, B and C (rate parameter - δ).

FFL-1 model

$$\frac{dS}{dt} = -\frac{S}{S + S_A} \times \frac{A}{A + A_S} \tag{3.1}$$

$$\frac{dA}{dt} = k_A \times \frac{S}{S + S_A} - \delta \times A \tag{3.2}$$

$$\frac{dB}{dt} = k_B \times \frac{A}{A + A_B} - \delta \times C \tag{3.3}$$

$$\frac{dC}{dt} = k_C \times \frac{1}{A + A_C} \times \frac{B}{B + B_C} - \delta \times C \tag{3.4}$$

Viral life cycle model We also consider a deterministic model describing the dynamics of levels of various viral molecules, like viral RNA species, viral proteins and viral particles present in a cell, infected by positive sense RNA virus [4].

SI S4 Data generation for fitting

Data used for fitting were computationally generated by simulating the model (on MATLAB). We assume values for the model parameters (Ground truth value, GT) and initial conditions, and simulate the model. The relevant model predictions sampled at the time points of interest constitute the data.

To emulate measurement error, we pick data points from a distribution centered around the model prediction. For example, suppose the model prediction for variable, V at time t, is $V(t) = V_1$. A corresponding noisy data would be $V_N(t) = V_2 \; Normal[V_1, \sigma]$, where $Normal[a, b]$ is a normal distribution with mean a and variance b^2.

We consider two dynamical (biological) systems to demonstrate various aspects of ABC-FAR, the parameter estimation algorithm developed. We consider (a) a two-species prey-predator system, and (b) a coherent feed forward (type-1) loop, described in the previous section.

Table S1: **Bounds used during fitting and the GT values used for data generation**

Parameter	Lower bound	Upper bound	$GT^{\#}$
**Lotka-Volterra model**			
$\log_{10}(a)$	-1	1	$\log_{10}(1)$
$\log_{10}(b)$	-2.5	0	$\log_{10}(0.02)$
$\log_{10}(c)$	-1	1	$\log_{10}(0.25)$
$\log_{10}(\delta)$	-2	0	$\log_{10}(0.3)$
dummy	0	1	
**FFL-1 model**			
$\log_{10}(k_A)$	-1	2	$\log_{10}(2.5)$
$\log_{10}(S_A)$	-1	2	$\log_{10}(3)$
$\log_{10}(A_S)$	-1	2	$\log_{10}(5)$
$\log_{10}(\delta)$	-2	1	$\log_{10}(0.1)$
$\log_{10}(k_B)$	-1	2	$\log_{10}(2.5)$
$\log_{10}(k_C)$	-1	2	$\log_{10}(5)$
$\log_{10}(A_B)$	-1	2	$\log_{10}(15)$
$\log_{10}(A_C)$	-1	2	$\log_{10}(2)$
$\log_{10}(B_C)$	-1	2	$\log_{10}(3)$
dummy	-1	2	
**GRN model**			
δ	-2		0.1
r_{base}	-3		0.05
k_1	$\log_{10}(0.3)$	$\log_{10}(30)$	1
k_2	$\log_{10}(0.3)$	$\log_{10}(30)$	2
k_3	$\log_{10}(0.3)$	$\log_{10}(30)$	3
k_4	$\log_{10}(0.3)$	$\log_{10}(30)$	4

For description of the model parameters refer to the discussion on corresponding models. $GT^{\#}$ refers to the ground truth values which was used to generate synthetic data.

Table S2: **Bounds used during fitting and the GT values used for data generation**

Parameter	Lower bound	Upper bound	GT#
**Viral life cycle model**			
**Free parameters**			
$\log_{10}(k_t)$	0	3	$\log_{10}(20)$
$\log_{10}(k_c)$	-5	-2	$\log_{10}(0.001)$
$\log_{10}(\tau_F)$	0	2	$\log_{10}(5)$
$\log_{10}(k_r)$	0	2	$\log_{10}(3)$
$\log_{10}(k_e)$	-2	1	$\log_{10}(0.6)$
$\log_{10}(N_C)$	1	4	$\log_{10}(250)$
$\log_{10}(k_a)$	-10	- 2	-7
dummy	0	1	
**Fixed parameters**			
μ_R			0.25
μ_P)			0.11
μ_V			0.06
η_S			180

For description of the model parameters refer to the discussion on corresponding models. GT# refers to the ground truth values which was used to generate synthetic data.

Table S3: **Simulation conditions to generate synthetic data**

**Lotka-Volterra model**	
Simulation time	0 - 30
Initial conditions	Prey population size: 75
	Predator population size: 20
**FFL-1 model**	
Simulation time	0 - 150
Initial conditions	We start with all model variables set to zero
	except the level of S which was set to 20.
**Three gene repressilator model**	
Simulation time	0 - 72 or 0 - 120
Initial conditions	We start with all model variables set to zero
	except the level of A which was set to 5.
**Viral life cycle model**	
Simulation time	0 - 48
Initial conditions	We start with all model variables set to zero
	except the level of cytoplasmic RNA (R_{cyt}) which was set to 1.

SI S5 Correlation-based statistics corresponding to selected parameter values

We posit that PnI(p, q) captures practical non-identifiability between the pth and the qth model parameters. The presence of correlation would indicate that changes in predictions of interest (in the context of data) due to perturbations in the pth parameter can be compensated via appropriate changes in the qth parameter, and vice-versa. For example, if PnI(p, q) is positive, then perturbation to predictions of interest due a decrease in value of pth parameter would be buffered by a decrease in value of qth parameter. Since this is Bayesian-based measure, we call it Bayesian Practical Identifiability. Note that the correlations would primarily capture local and linear effects.

Additionally we can also look at correlation between selected parameter values and corresponding χ^2 values. Let ConvCorr(p) evaluates the correlation between the value of the pth parameter in the selected combination and the corresponding χ^2 value. A positive (negative) correlation suggests that among the selected values of the parameter, the ones with lower (higher) value correspond to predictions with higher (lower) deviation from data. Thus the presence of correlation indicate that further refinement in (the centrality measure of) the posterior is needed. Note that the correlation between χ^2 and a model parameter is a local property, conditional on the other model parameter values being fixed. We can look at a more global measure - TCC. TCC denotes the total magnitude of correlation between selected parameter values and χ^2 summed over all model parameter. If TCC decays to zero, then each term in the sum goes to zero, suggesting convergence to a minima for each of the parameters. Simultaneous local convergence in turn implies global convergence in (the centrality measure of) posterior estimation. Thus we posit the decay of TCC to zero to indicate convergence of model fitting. Since TCC based convergence is Bayesian computation based, we refer to it as a Bayesian correlation-convergence criterion.

We borrow the same notations as used in the main text. $\vec{\theta}^1, \vec{\theta}^2, \ldots, \vec{\theta}^M$ are the M parameter combinations selected based on their χ^2 values. Let θ_p^i denote the value of the pth model parameter in the ith combination. Further let χ_i^2 denote the χ^2 statistics corresponding to the ith parameter combination, $\vec{\theta}^i$. The correlative measures are evaluated as:

$$\text{ConvCorr(p)} = \frac{M\sum_{i=1}^{M}\left(\theta_p^i.\chi_i^2\right) - \left(\sum_{i=1}^{M}\theta_p^i\right)\left(\sum_{i=1}^{M}\chi_i^2\right)}{\sqrt{M\sum_{i=1}^{M}(\theta_p^i)^2 - (\sum_{i=1}^{M}\theta_p^i)^2}\sqrt{M\sum_{i=1}^{M}(\chi_i^2)^2 - (\sum_{i=1}^{M}\chi_i^2)^2}}$$

$$\text{TCC} = \sum_{p=1}^{k}|\text{ConvCorr(p)}|$$

SI S6 Efficiency of parameter estimation algorithm

We evaluate (a) minimum/maximum χ^2 statistics, and (b) Effective Acceptance Rate (EAR), to fairly compare the performance of different schema of ABC-FAR with each other and with the reported performance of ABC-SMC. Minimum and maximum χ^2 statistics looks at the minimum and maximum of the χ^2 values corresponding to the parameter combinations selected at every iteration of the estimation procedure. Central tendencies, like mean or median, of the (selected) χ^2 values can also be used. These χ^2 statistics indicate the goodness of fit, or how well the model predictions mimic the data used for fitting. On the other hand, the Effective Acceptance Rate (EAR) looks at the (inverse of the) cumulative computational complexity. EAR is proportional to the total number of parameter combinations evaluated or the total number of times the model was simulated.

$$\text{EAR} = \frac{\text{FAR}}{\text{Iteration index}} = \frac{\text{M}}{\text{N} \times \text{Iteration index}} = \frac{\text{Number of parameter combinations selected}}{\text{Total number of parameter samples evaluated}},$$

where N and M are the numbers of parameter combinations sampled and selected in an iteration respectively.

An algorithm or scheme leads to a good/accurate fit if it can identify parameter combinations corresponding to low χ^2 values. Comparing schemes that reach a particular threshold of χ^2 statistics, the one with higher EAR (that is, less computational cost) is considered more efficient than the other. Comparing the different schemes of our algorithm was straightforward as we use the same χ^2 function and identical value of M (the number of parameter combinations selected in an iteration). However, we have to normalize for rejection criterion and differences in the χ^2 function while comparing different algorithms. The ABC-SMC study reports their performance for a single iteration implementation along with the performance of an optimized realization of their algorithmic parameters. We consider the following normalization,

$$\chi^2_n = \chi^2_{SMC} \times \frac{S_{FAR}}{S_{SMC}}$$

where χ^2_n is the normalization of χ^2_{SMC} (from ABC-SMC paper) based on S_{FAR} and S_{SMC} which are the χ^2 statistics corresponding to single iteration implementation of ABC-FAR and ABC-SMC algorithms respectively. To normalize for different rejection criteria, we calculate S_{FAR} imposing a FAR value of 7×10^{-4}, so that the EAR corresponding to both S_{FAR} and S_{SMC} are same.

SI S7 Compatibility of ABC-FAR to utilize system-structure to improve convergence

In the previous section we saw how algorithm-based strategies affect efficiency of parameter estimation. In some situations, we can also utilize system-specific knowledge to speed up and/or improve convergence, for example, when we have partial knowledge about parameter value distribution or the mathematical structure of the model allows us to define the fitting problem cleverly. We demonstrate the compatibility of our program to exploit such information to speed up and improve convergence. We demonstrate how we can do so for the FFL1 system, described in the previous section.

According to the model, the dynamics of levels of S and A are influenced only by each other. Thus we can focus on the sub-model and data associated with only their dynamics to estimate the associated parameters - $\log_{10}(\mathrm{k}_A)$, $\log_{10}(\mathrm{S}_A)$, $\log_{10}(\mathrm{A}_S)$ and $\log_{10}(\delta)$). We label this sub-problem as 'H1', where we ignore the parts of the model associated with dynamics of B and C. Next we observe that dynamics of B is affected by levels of S and A, but not by the level of C. Thus we define sub-problem 'H2', where we augment the sub-problem H1 with the dynamics of B and the additional parameters associated with it - $\log_{10}(\mathrm{k}_B)$ and $\log_{10}(\mathrm{A}_B)$). The posterior estimated in H1, for the first four parameters can be used as prior in H2. After H2, we finally consider the entire model in sub-problem 'H3'. For all the older parameters, we use the posterior estimated for the parameters in H2, as the prior for respective parameters in H2. We denote this strategy of defining sub-problems as Hierarchical calibration (hCal). We compare its efficiency against the one-shot calibration (oCal) strategy where we fit the entire model at a single go.

In this analysis, parameter values in log scale to explore a large dynamical range. Also, whenever a parameter is estimated for the first time in either of the calibration strategies, we use a uniform prior. So for hCal, we use a uniform prior for (a) $\log_{10}(\mathrm{k}_A)$, $\log_{10}(\mathrm{S}_A)$, $\log_{10}(\mathrm{A}_S)$ and $\log_{10}(\delta)$) in H1, (b) $\log_{10}(\mathrm{k}_B)$ and $\log_{10}(\mathrm{A}_B)$) in H2, (c) $\log_{10}(\mathrm{k}_C)$, $\log_{10}(\mathrm{A}_C)$) and $\log_{10}(\mathrm{B}_C)$ in H3; we use uniform priors for all the parameters in the oCal strategy. We use two iterations each for H1 and H2, four for H3, and eight for oCal; thus a fair comparison can only be made after the fifth iteration when the entire model is considered and χ^2 functions used in both the strategies are identical. Other algorithm options are kept identical: FAR = 10%, HDUS, parallel execution. To compare efficiency we look at the cumulative execution of time (cTime) and χ^2 values for selected parameter combinations.

We see that both the calibration strategies fits the model to data (SFigure S7), but hCal is more efficient and accurate compared to oCal. For example, fifth iteration onwards (a) the χ^2 values corresponding to the selected parameters, and (b) the cumulative execution time, are lower for hCal compared to oCal. Actually, hCal outperforms oCal by almost an iteration in both these measures. cTime corresponding to the seventh iteration of hCal compares with that of the sixth iteration of oCal, whereas χ^2 distribution corresponding to the seventh iteration of hCal is as good as that of the eighth iteration of

oCal (SFigure S7). The better performance of hCal can be attributed to (a) fewer calculations required for simulation and χ^2 evaluation in H1 and H2, (b) lower dimensionality of parameter space for H1 and H2, and (c) propagation of information about parameters from one sub-problem to next.

Our algorithm was conveniently adapted and easily set-up to execute the hCal strategy. We didn't have to define different sets of parameter rejection threshold for the three sub-problems associated with hCal, a convenient advantage over the typically used ABC-based parameter estimation algorithms.

Table S4: **Top three designs of each modality (based on estimation error)**

Design ID	Time points of measurements (hours)	Estimation error (a.u.)
PARSEC #71	[3 12 24 33 36 54]	0.1
PARSEC #95	[9 15 27 36 42 60]	0.1
PARSEC #4	[6 18 33 39 57 69]	0.11
RANDOM #5	[6 9 15 36 42 60]	0.12
RANDOM #62	[6 15 24 36 39 69]	0.12
RANDOM #4	[15 27 36 45 54 72]	0.14
anti-PARSEC #11	[3 6 9 15 18 21]	0.57
anti-PARSEC #16	[33 60 63 66 69 72]	0.8
anti-PARSEC #2	[15 18 24 27 30 33]	0.81

These designs correspond to the analysis presented in Figure 4 of main text. Estimation error is in arbitrary units (see Methods section in the Main text)..

SI S8 The predicted designs

The table lists the top designs from each class.

Supplementary figures

Rationalised experiment design for parameter estimation with sensitivity clustering

Harsh Chhajer, Rahul Roy

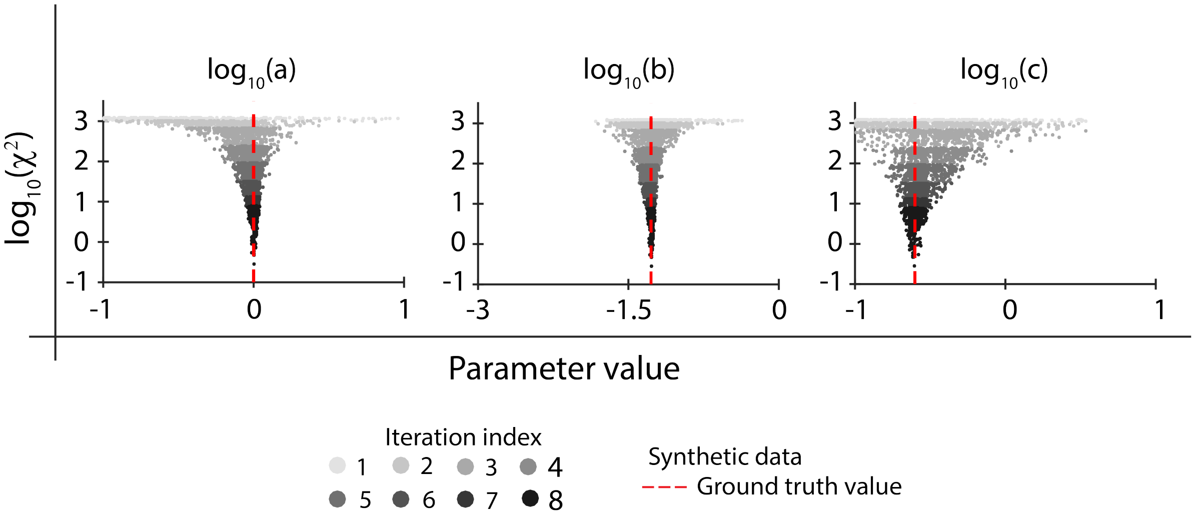

Figure S2: Scatter-plot showing the the parameter values selected and the corresponding χ^2 value, in each iteration of fitting the Lotka-Volterra model. The values of iteration index are represented by shades of gray. Here the prey-predator model is fit to the synthetic data, generated using the parameter values denoted as 'Ground truth' values, depicted by the red dashed line.

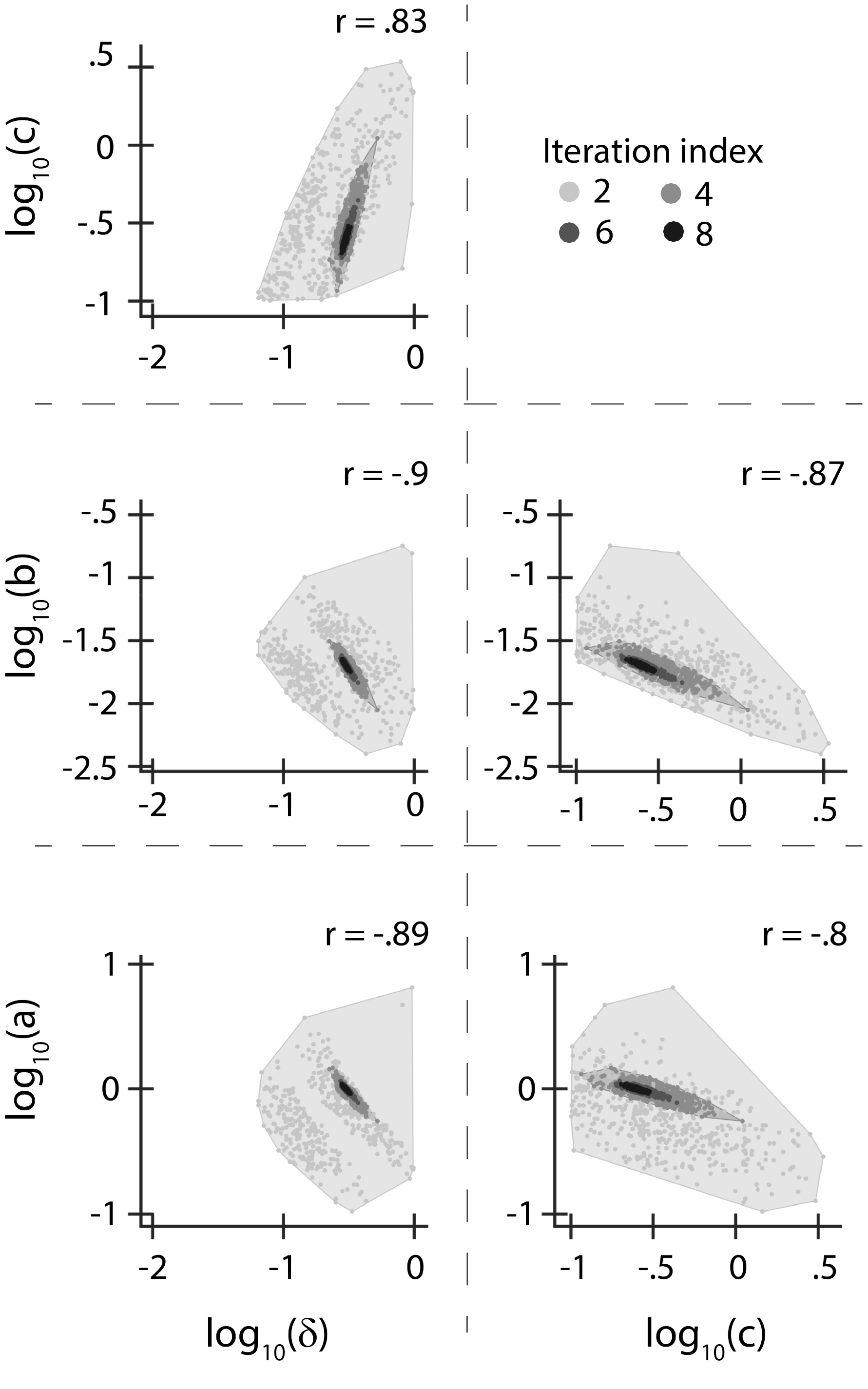

Figure S3: 2D scatter-plots (and the convex hull boundary) of parameter combinations selected in the second, fourth, sixth and eighth iterations of fitting the Lotka-Volterra model. The iterations are represented by the shades of gray. r-value shown in the top-right corner for each sub-figure shows the correlation between the values, of corresponding parameter-pair, selected in the final (eighth) iteration.

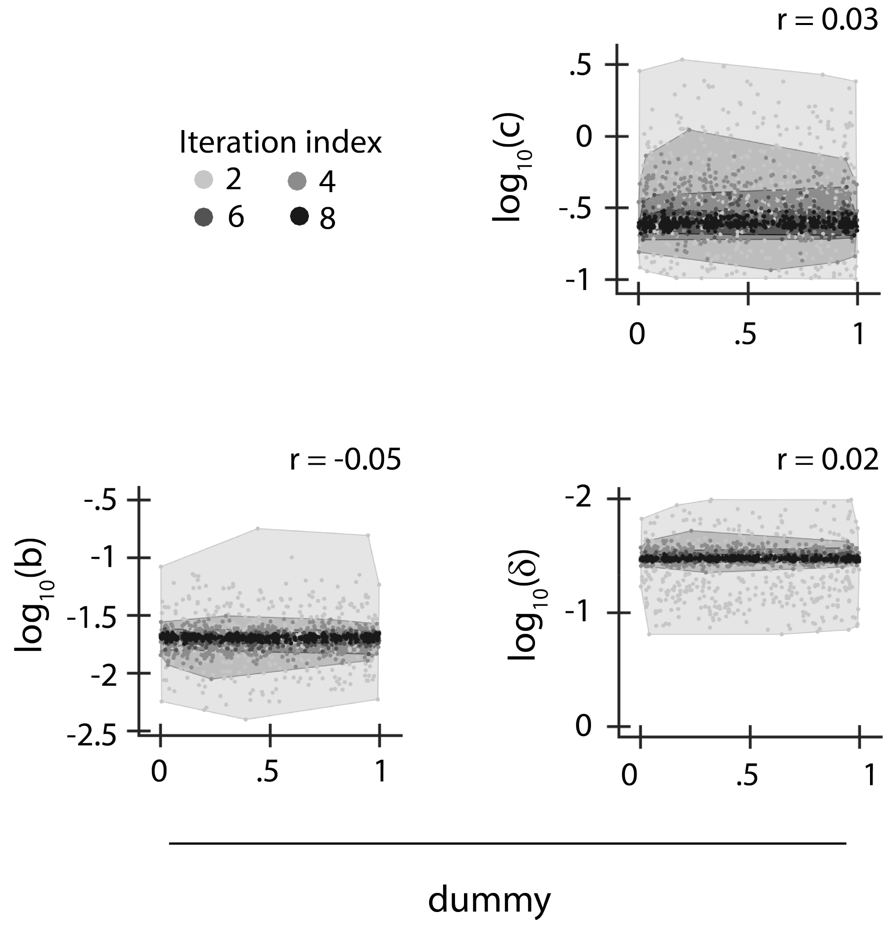

Figure S4: 2D scatter-plots (and the convex hull boundary) of parameter combinations selected in the second, fourth, sixth and eighth iterations of fitting the Lotka-Volterra model. The iterations are represented by the shades of gray. r-value shown in the top-right corner for each sub-figure shows the correlation between the value of corresponding parameter and dummy parameter value selected in the final (eighth) iteration.

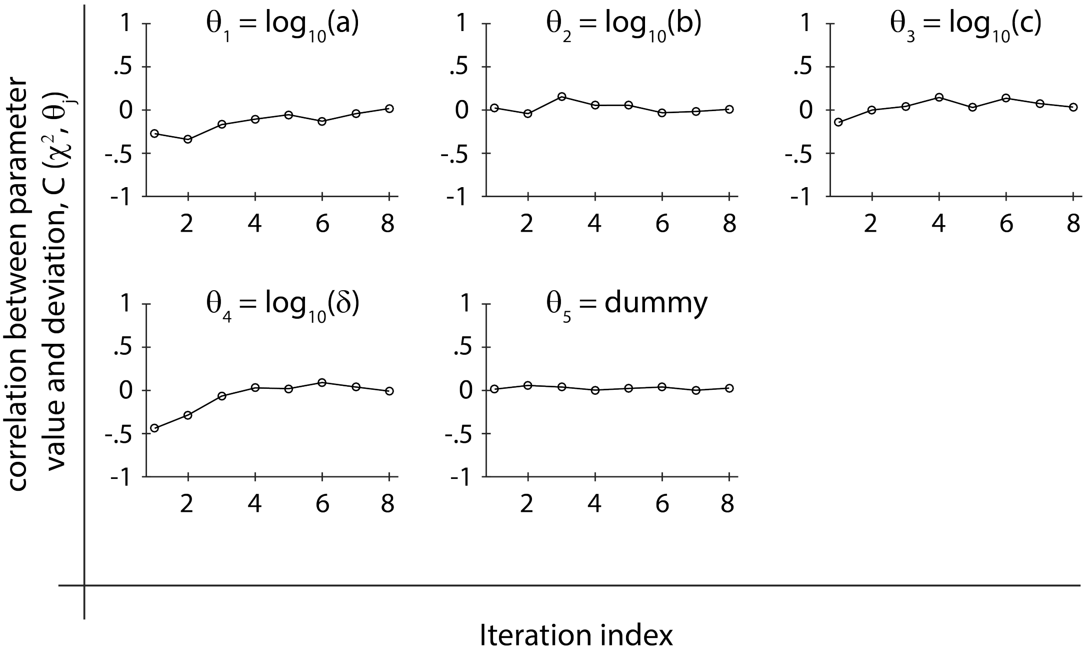

Figure S5: Variation of the correlation between the χ_2 and the values of the parameters, selected after every iteration, while fitting the Lotka-Volterra model.

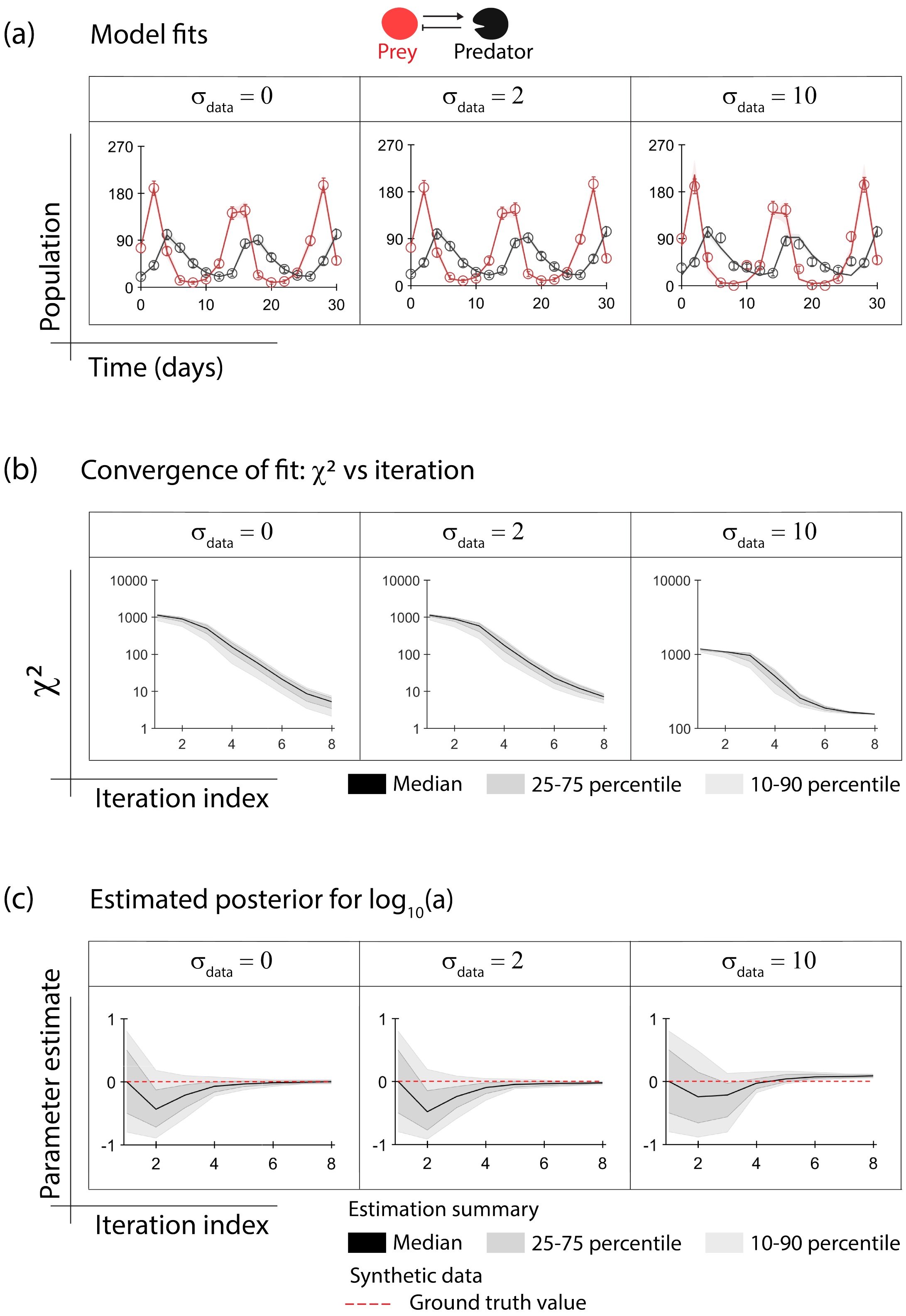

Figure S6: **Model fitting and parameter estimation using noisy data**
Data used for fitting the Lotka-Volterra model was augmented with noise to mimic measurement error (σ_{data}) in measuring population size. (a) The model predictions due to parameter combinations selected at the final iteration (thin lines) explain the noisy data (open circle) well. The thick lines show the mean of predictions due to the selected parameter combinations. (b) Statistics for the distribution of χ^2 corresponding to the parameter combinations selected at each iteration. (c) Statistics for the posterior of $\log_{10}(a)$ estimated at each iteration.

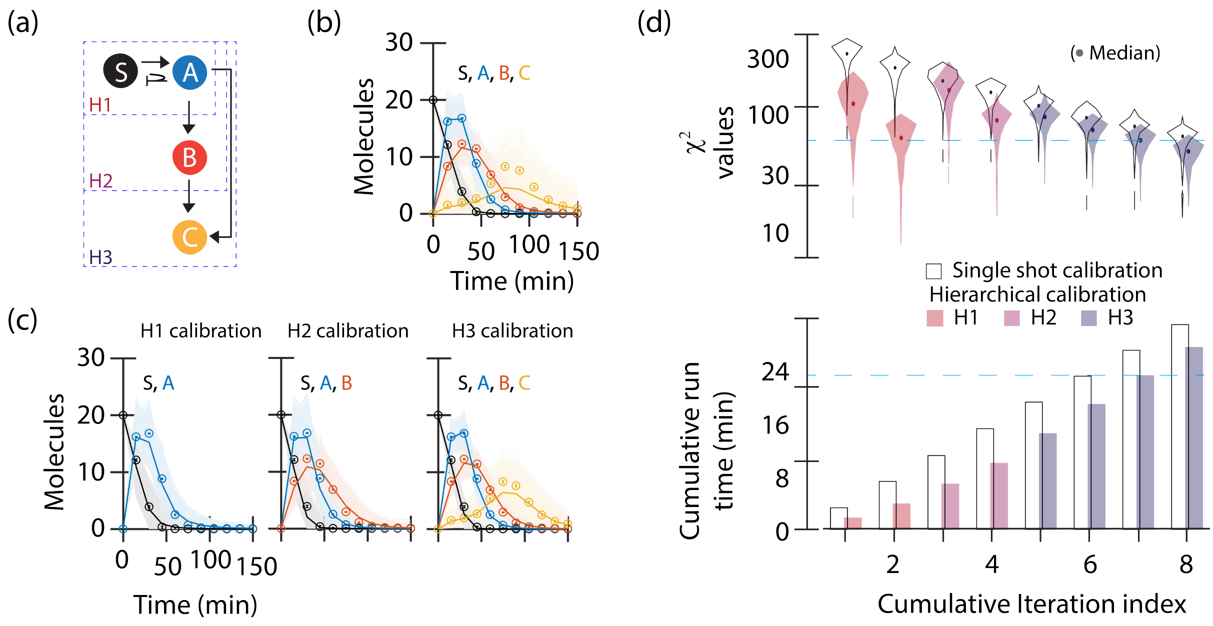

Figure S7: **Exploiting model structure for more efficient model fitting**
(a) Schematic of the Feed-forward loop (FFL) system. (b) Model fitting due to oCal strategy is shown. (c) Model fitting after H1 (left), H2 (middle) and H3 (right) are shown. In subfigs b and c, thin lines represent model predictions due to individual parameter combinations selected at the final iteration, the thick lines track the mean of predictions over selected parameter combinations and open circles represent the data used for fitting. (d) Efficiency of oCal and hCal strategies are compared in terms of accuracy (top) and time complexity (bottom).

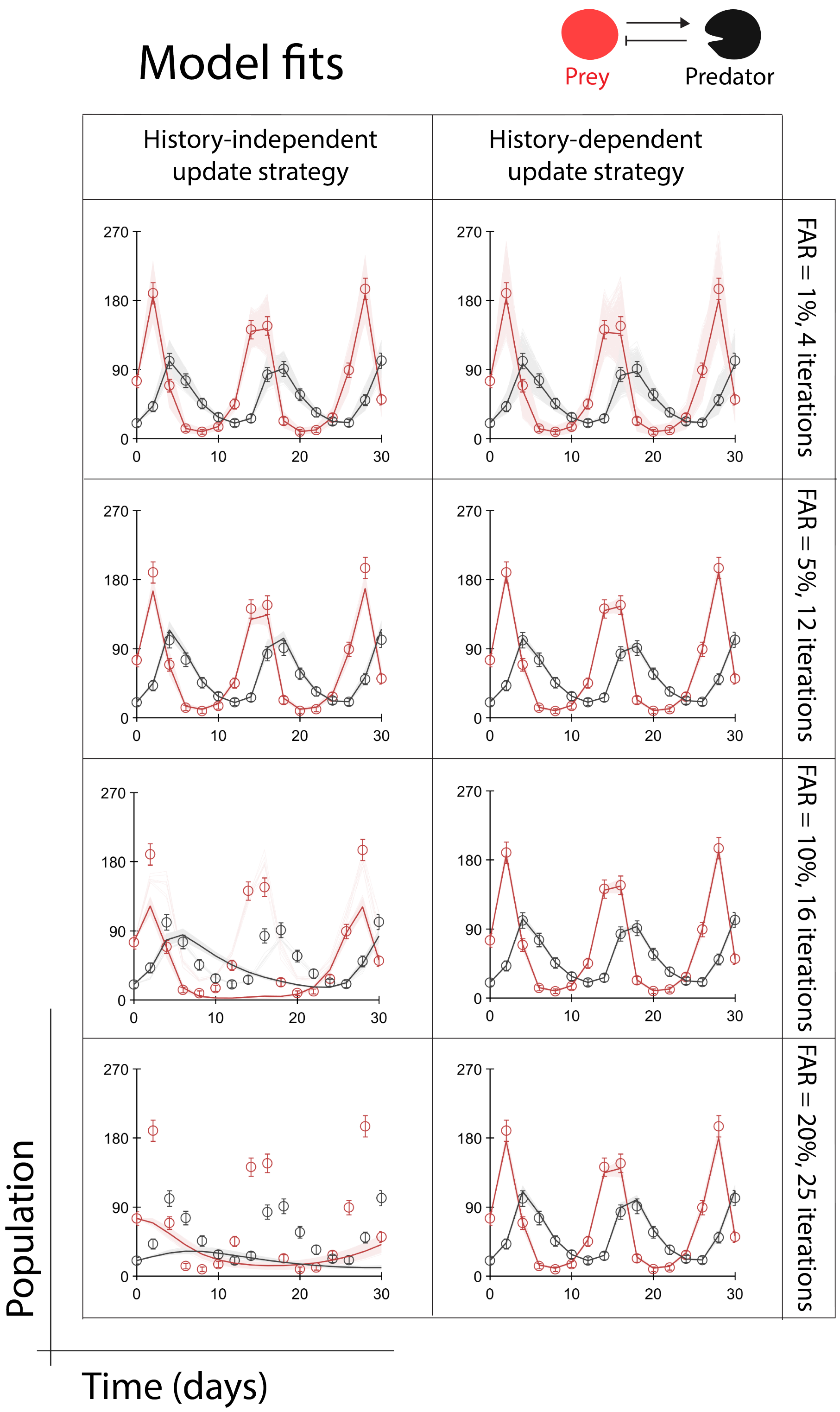

Figure S8: **Tuning algorithmic options** Here we see how the Lotka-Volterra model fitting is affected when we vary the update strategies (History-independent update strategy and History-dependent update strategy) and FAR values. Here the thin lines represent model predictions due to individual parameter combinations selected at the final iteration, the thick lines track the mean of predictions over selected parameter combinations and open circles represent the data used for fitting.

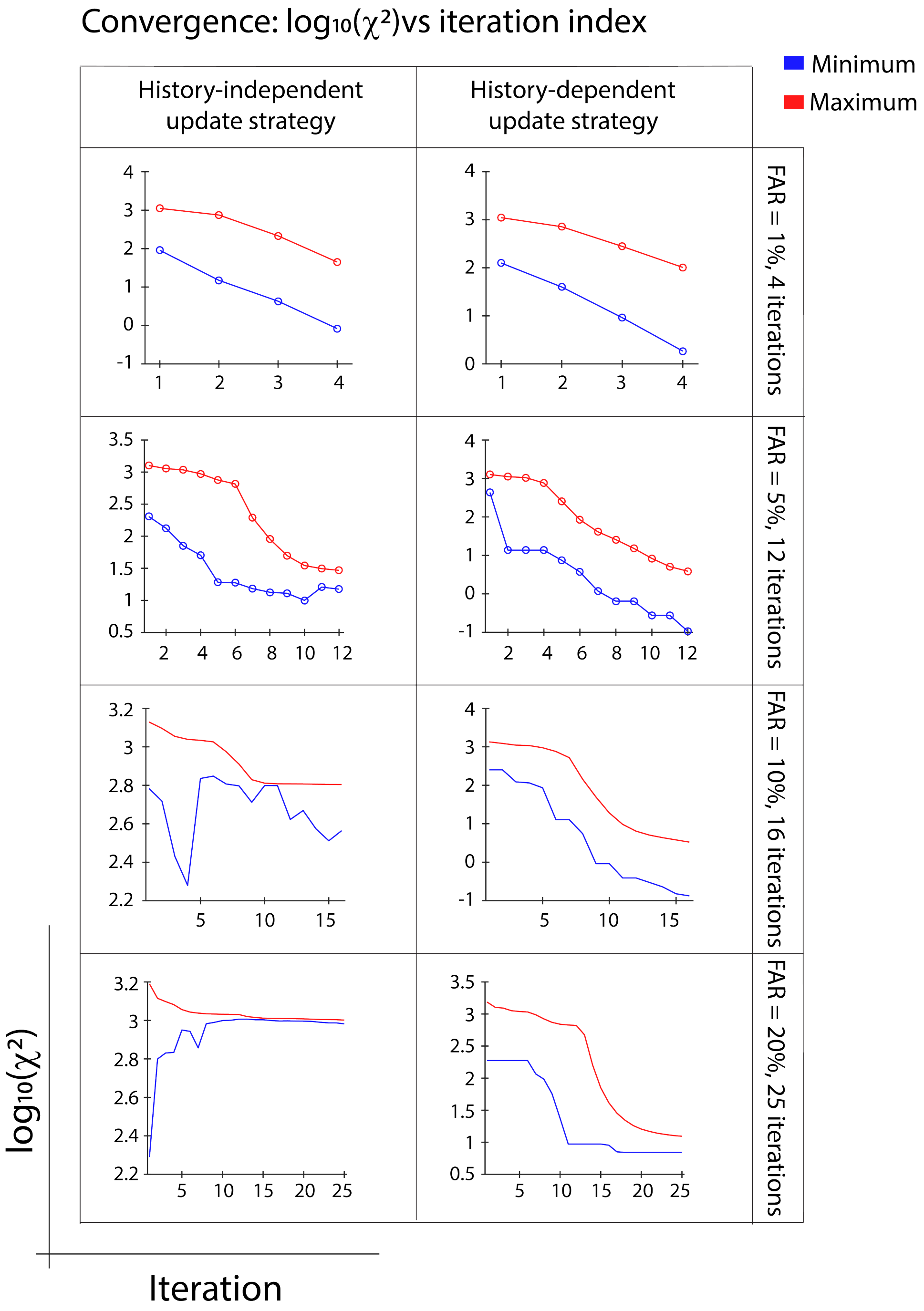

Figure S9: **Tuning algorithmic options** Here we see how the Lotka-Volterra model fitting is affected when we vary the update strategies (History-independent update strategy and History-dependent update strategy) and FAR values. The minimum (blue) and the maximum (red) of the χ^2 values corresponding to the parameter combinations selected in every iteration is plotted.

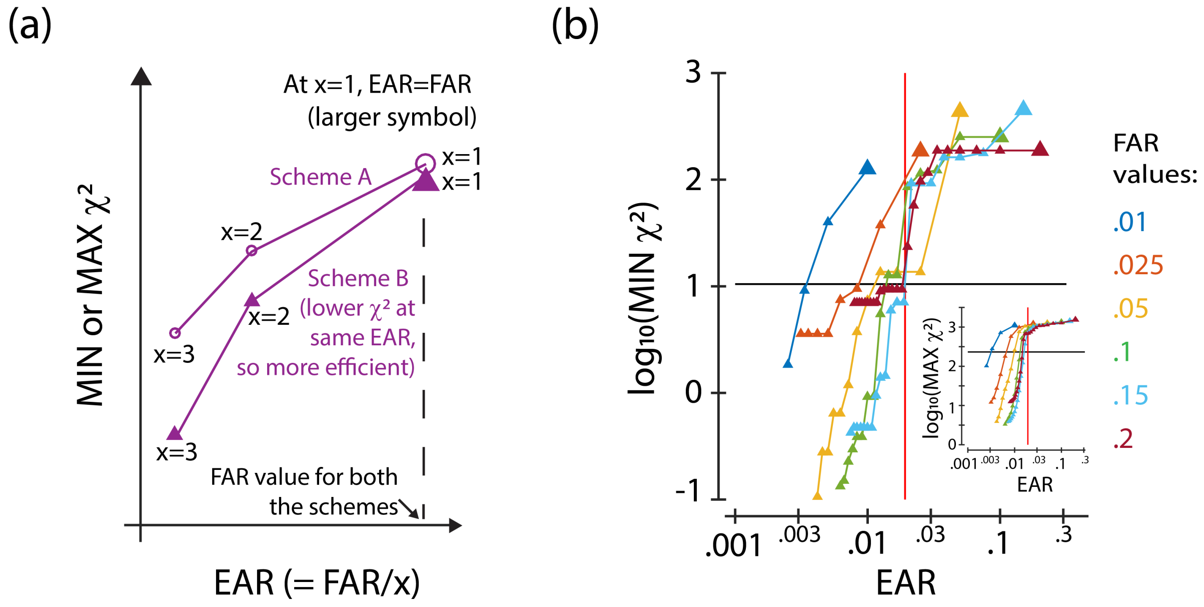

Figure S10: **Accuracy and efficiency of the parameter estimation algorithm**
(a) To characterize the efficiency of the estimation process we look at accuracy and computational cost. Accuracy is inferred by the minimum of χ^2 values corresponding to the selected combinations; mean or maximum value can also be considered. Computational cost is measured by the total number of parameter combinations sampled and simulated. Thus the cost is inversely proportional to Effective Acceptance Rate (EAR), as the number of parameter combinations selected in each iteration is kept constant throughout the analysis. An efficient process would have a lower χ^2 statistic at a given EAR value, or high EAR when we fix the χ^2 statistic. (b) We characterize the efficiency for each iteration of a parameter estimation process to fit the Lotka Volterra model to computationally generated data, using ABC-FAR with different values of FAR. We see that χ^2 statistic initially decreases with decrease in EAR (faster for higher FAR value) and then saturates (at higher EAR for higher FAR value). The efficiency of our algorithm compares well with that reported for ABC-SMC, shown in the figure by the intersection of black and red solid lines.

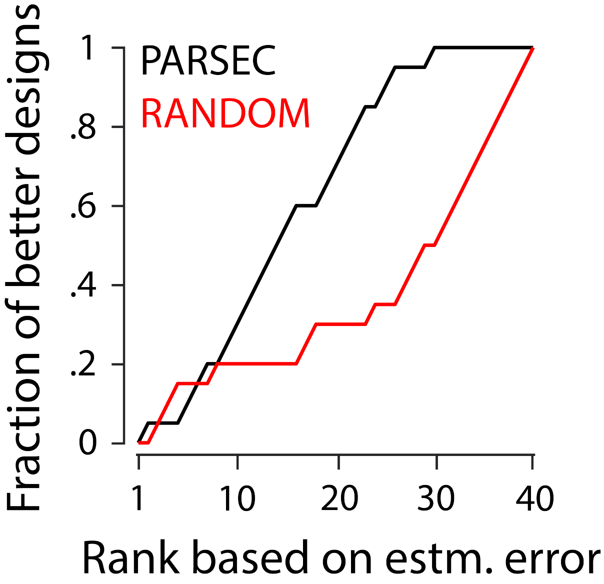

Figure S11: **Ranking the PARSEC and random designs for the repressillator system**
Twenty PARSEC and 20 random designs for the system corresponding to Figure 4 (main text) are ranked based on the predicted estimation error, and plotted here,

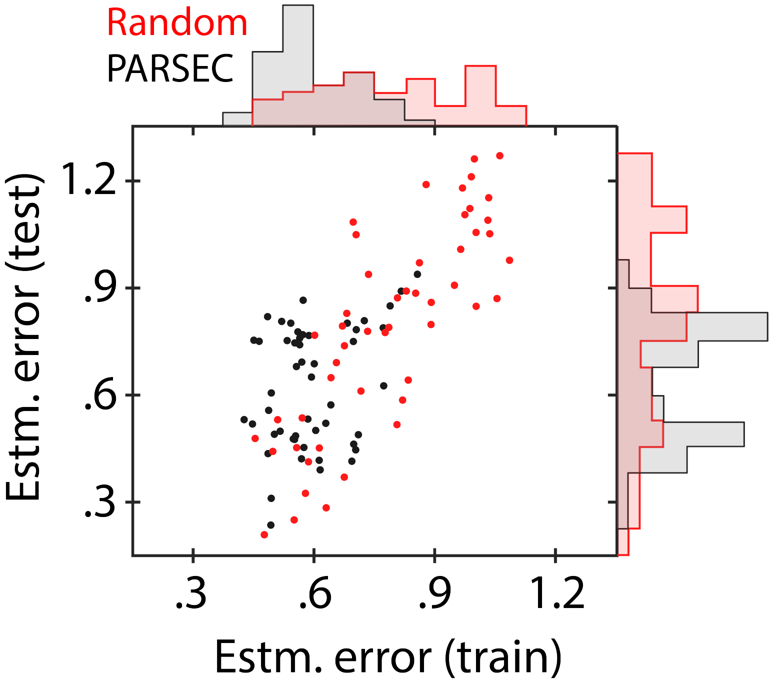

Figure S12: **Robustness of PARSEC(k)**
Here we consider a nine-fold uncertainty characterized by $log_2(k_1/k_1^0) \in U[-log_2(3), log_2(3)]$ and $log_2(k_2/k_2^0) \in U[-log_2(3), log_2(3)]$. where $U[a, b]$ denotes a uniform distribution bounded by a and b. PSI are evaluated at nine training samples (Θ^k) representing this distribution, are used to construct the PARSEC-PSI vectors. These are grouped using k-means clustering to inform the selection of PARSEC(k) designs. 100 PARSEC(k) and 100 random designs are evaluated for estimation accuracy at the training samples (Training analysis) and at four new test samples (Ω^k, test analysis). Sampling was done via Latin Hypercube Sampling. The average estimation error across the training samples and the test samples are evaluated as train and test estimation errors respectively. The corresponding marginals are also shown. The plot highlights that PARSEC(k) designs are more informative. The 2D-plot of the train and test estimation errors verify the equivalence of performance of the best few PARSEC(k) designs against stochastic sampling, indicating their robust performance.

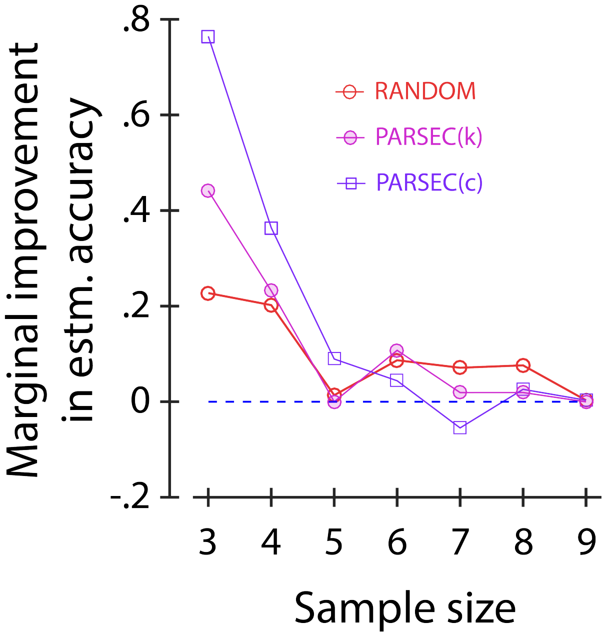

Figure S13: **Marginal gain in estimation accuracy due to increase in experiment sample size** (a) We plot the dynamics of the three-gene repressilator system for the parameter combination used for the design. (b) One of the realizations of c-means clustering of the measurement candidates for cluster multiplicity ranging from two to nine is shown. Clusters are identified using different colors.

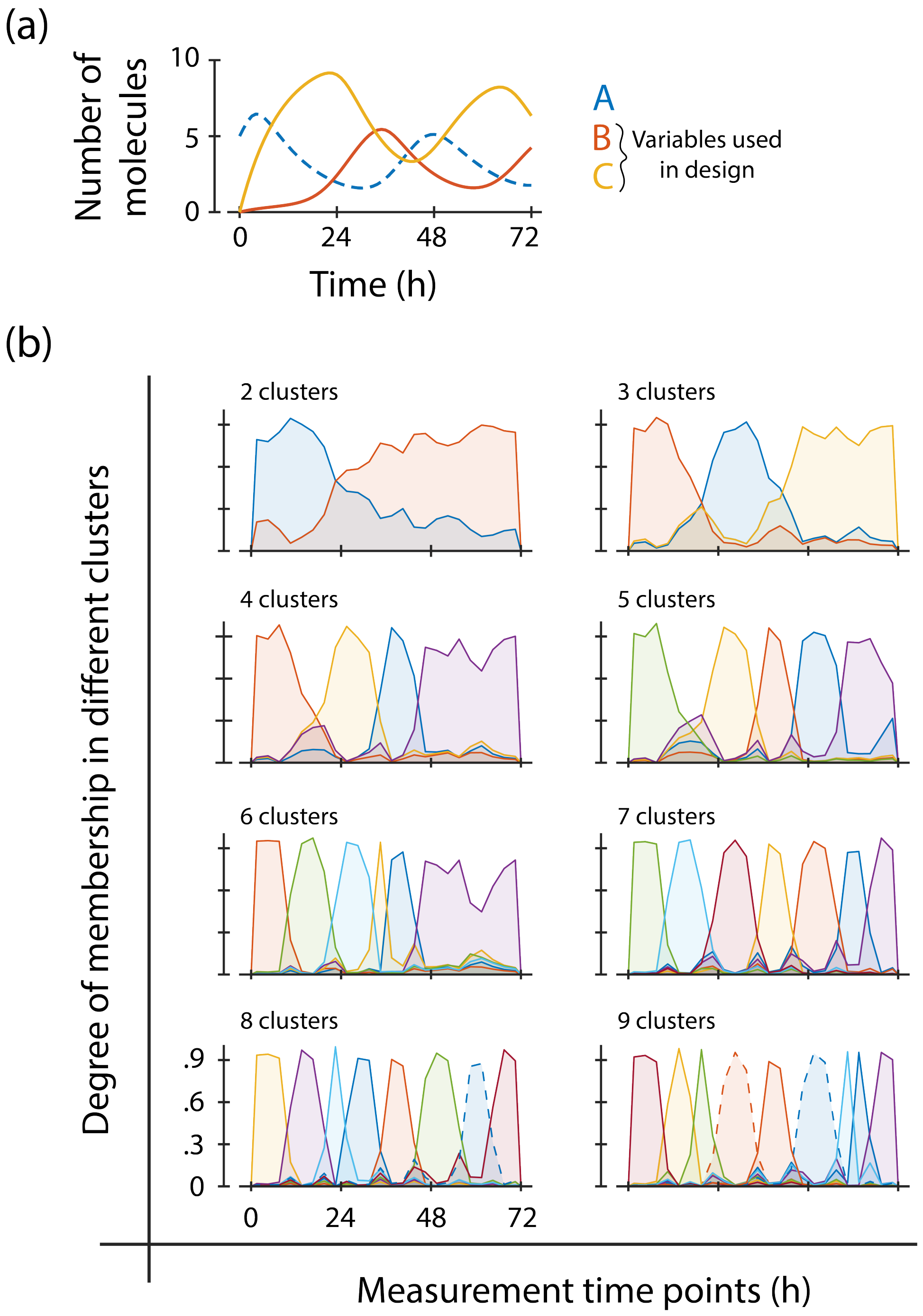

Figure S14: **Partitioning via c-means clustering algorithm**
(a) We plot the dynamics of the three-gene repressilator system for the parameter combination used for the design. (b) One of the realizations of c-means clustering of the measurement candidates for cluster multiplicity ranging from two to nine is shown. Clusters are identified using different colors.

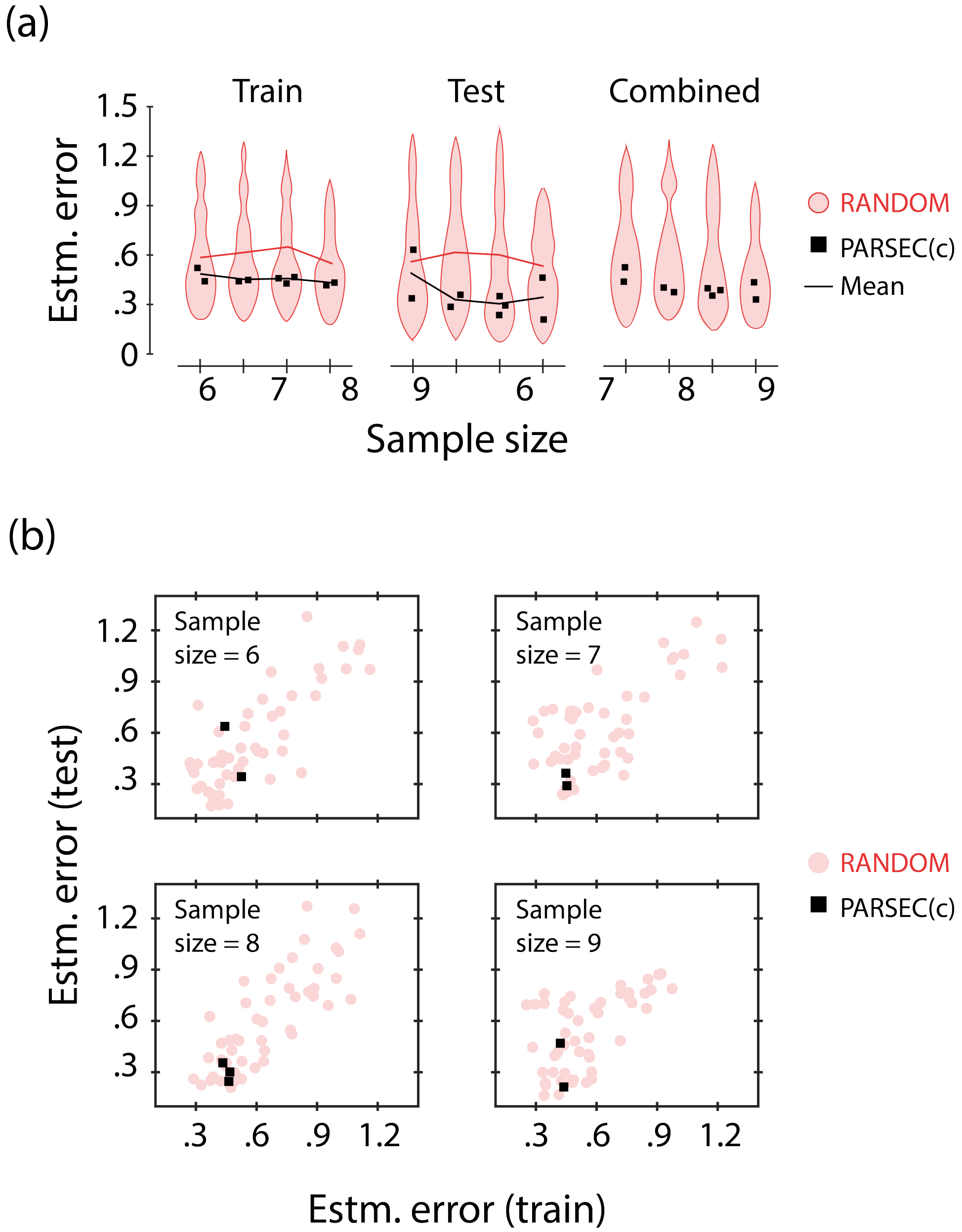

Figure S15: **Performance of generalist design constructed via PARSEC(c)**
We use PARSEC(c), which uses c-means clustering, to efficiently identify informative generalist designs according to the specifications used for the analysis summarized in Figure 5 (Main text). We also consider the same training and test samples as used there. The corresponding dynamics and a realization of the c-means clustering is shown in Supplementary Figure S16. (a) We plot the distribution of estimation error averaged across the training (Train statistics, left) and testing (Test statistics, right) samples for 50 random designs (red), and the estimation error for each of the unique PARSEC(c) designs (black) considered in the analysis. We also show the average statistics for the estimation error for the two modalities of design as a function of sample size. The average estimation error reduces with an increase in sample size. Although PARSEC(c) may miss the most informative designs, on average it identifies designs more informative compared to random designs with about 44 times fewer calculations. (b) The 2D-plot of the train and test estimation errors verify the equivalence of performance of PARSEC(c) against stochastic sampling, indicating their robust performance.

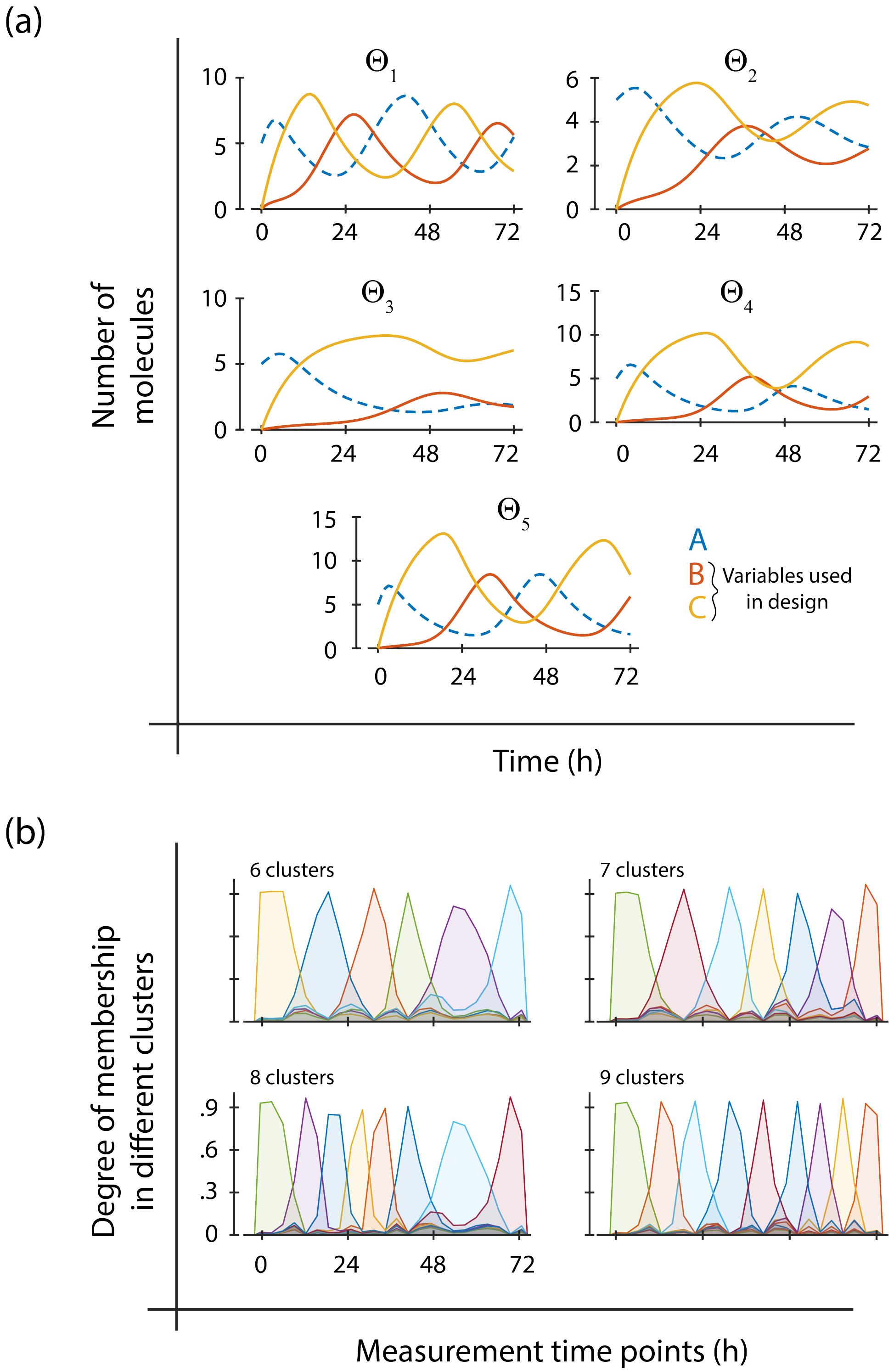

Figure S16: **Dynamics and c-means clustering involved in constructing generalist designs using PARSEC(c)**
(a) We plot the dynamics of the three-gene repressilator system for the five training samples used in the design construction. (b) One of the realizations of c-means clustering of the measurement candidates for cluster multiplicity ranging from six to nine is shown. Clusters are identified using different colors.

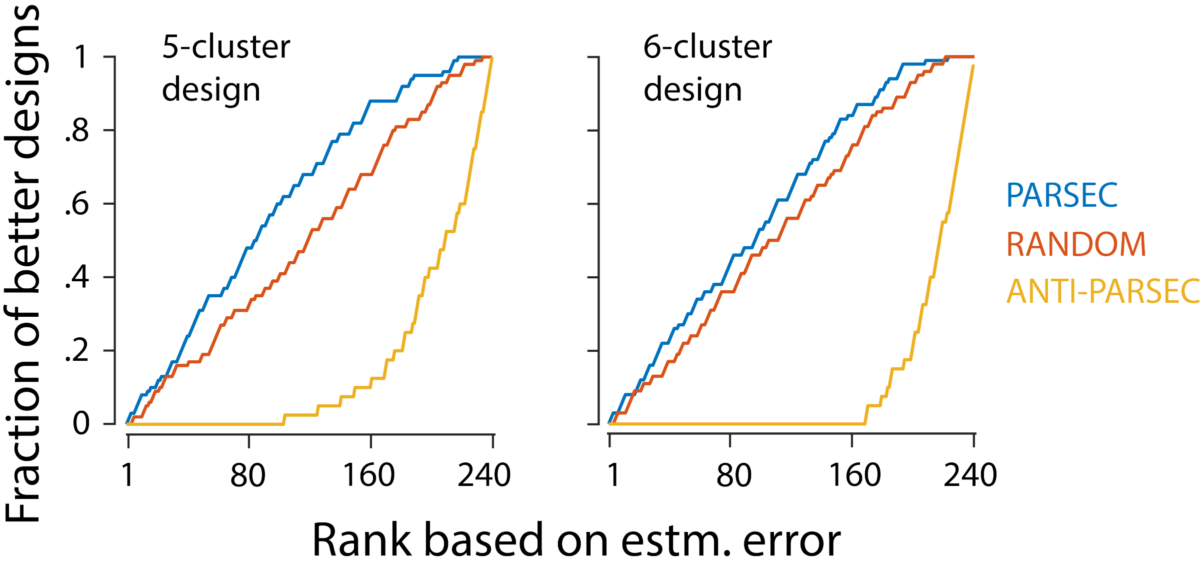

Figure S17: **Ranking the PARSEC and random designs for the viral life cycle system**
We predicted 100 PARSEC, 100 random, and 40 anti-PARSEC designs using our framework to characterize the viral life cycle parameters [4]. These designs are ranked based on the predicted estimation error as shown here. In the analysis, the set of feasible time points of measurements is 2, 4, 6, . . ., 48 hours post-infection. We see that PARSEC designs in general rank better, at which the levels of total viral (+)RNA ($R_{cyt} + R_{CM} + RC_{CM}$), dsRNA ($RC_{CM}$) and viral titre ($V_T$) can be measured simultaneously.

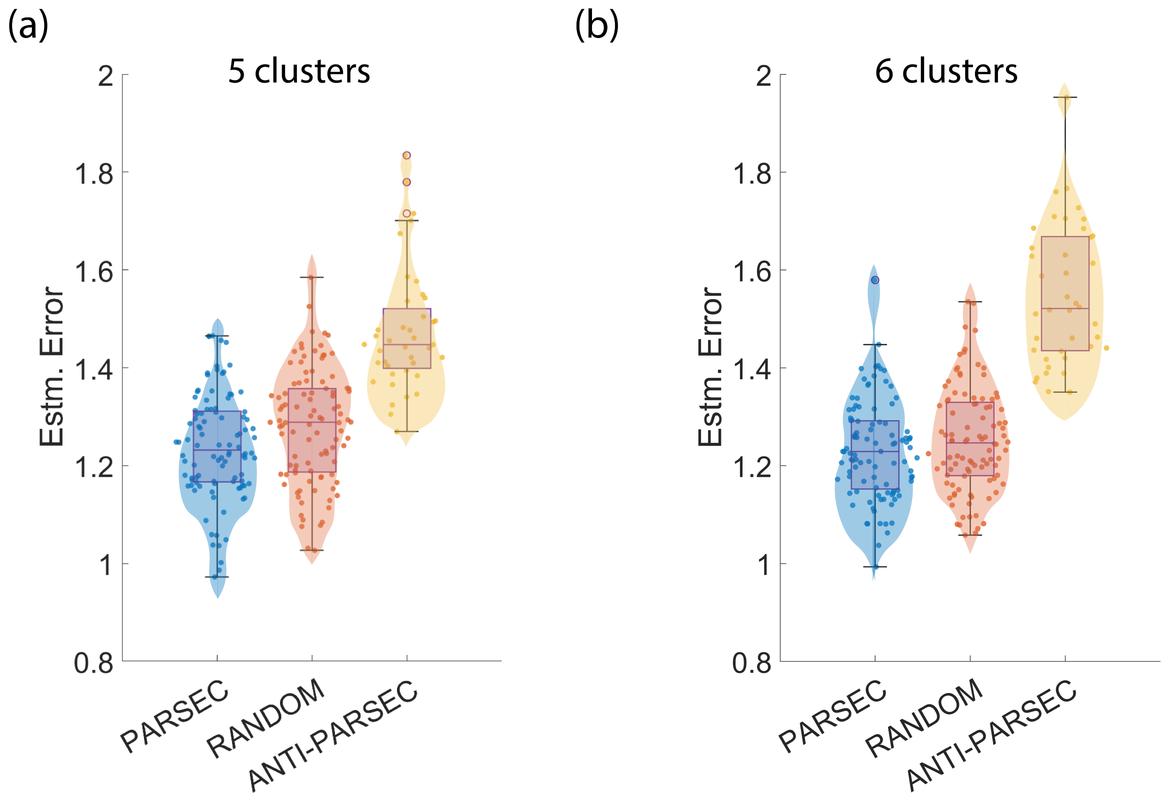

Figure S18: **Estimation error for the PARSEC and random designs for the viral life cycle system**

We predicted 100 PARSEC, 100 random, and 40 anti-PARSEC designs using our framework to characterize the viral life cycle parameters [4]. These designs are ranked based on the predicted estimation error as shown here. In the analysis, the set of feasible time points of measurements is 2, 4, 6, . . ., 48 hours post-infection. We see that PARSEC designs in general have lower estimation error , at which the levels of total viral (+)RNA ($R_{cyt} + R_{CM} + RC_{CM}$), dsRNA ($RC_{CM}$) and viral titre ($V_T$) can be measured simultaneously.

References

[1] Michael D McKay, Richard J Beckman, and William J Conover. A comparison of three methods for selecting values of input variables in the analysis of output from a computer code. *Technometrics*, 42(1):55–61, 2000.

[2] Scott Kirkpatrick, C Daniel Gelatt, and Mario P Vecchi. Optimization by simulated annealing. *science*, 220(4598):671–680, 1983.

[3] Tina Toni, David Welch, Natalja Strelkowa, Andreas Ipsen, and Michael PH Stumpf. Approximate bayesian computation scheme for parameter inference and model selection in dynamical systems. *Journal of the Royal Society Interface*, 6(31):187–202, 2009.

[4] Harsh Chhajer, Vaseef A Rizvi, and Rahul Roy. Life cycle process dependencies of positive-sense rna viruses suggest strategies for inhibiting productive cellular infection. *J R Soc Interface*, 2021.
